# A multi-scale model of gas transport in the lung to study heterogeneous lung ventilation during the multiple-breath washout test

**DOI:** 10.1371/journal.pcbi.1007079

**Published:** 2019-06-17

**Authors:** David Hasler, Pinelopi Anagnostopoulou, Sylvia Nyilas, Philipp Latzin, Johannes Schittny, Dominik Obrist

**Affiliations:** 1 Pediatric Respiratory Medicine, Department of Pediatrics, Inselspital, Bern University Hospital, University of Bern, Bern, Switzerland; 2 ARTORG Center for Biomedical Engineering Research, University of Bern, Bern, Switzerland; 3 Institute of Anatomy, University of Bern, Bern, Switzerland; 4 Department of Diagnostic, Interventional, and Pediatric Radiology, Inselspital, Bern University Hospital, Bern, Switzerland; Stanford University, UNITED STATES

## Abstract

The multiple-breath washout (MBW) is a lung function test that measures the degree of ventilation inhomogeneity (VI). The test is used to identify small airway impairment in patients with lung diseases like cystic fibrosis. However, the physical and physiological factors that influence the test outcomes and differentiate health from disease are not well understood. Computational models have been used to better understand the interaction between anatomical structure and physiological properties of the lung, but none of them has dealt in depth with the tracer gas washout test in a whole. Thus, our aim was to create a lung model that simulates the entire MBW and investigate the role of lung morphology and tissue mechanics on the tracer gas washout procedure. To this end, we developed a multi-scale lung model to simulate the inert gas transport in airways of all size. We then applied systematically different modifications to geometrical and mechanical properties of the lung model (compliance, residual airway volume and flow resistance) which have been associated with VI. The modifications were applied to distinct parts of the model, and their effects on the gas distribution within the lung and on the gas concentration profile were assessed. We found that variability in compliance and residual volume of the airways, as well as the spatial distribution of this variability in the lung had a direct influence on gas distribution among airways and on the MBW pattern (washout duration, characteristic concentration profile during each expiration), while the effects of variable flow resistance were negligible. Based on these findings, it is possible to classify different types of inhomogeneities in the lung and relate them to specific features of the MBW pattern, which builds the basis for a more detailed association of lung function and structure.

## Author summary

Obstructive lung diseases, like cystic fibrosis or primary ciliary dyskinesia, lead to inhomogeneous ventilation. The degree of observed inhomogeneity represents a clinical measure for the progression of the disease. The multiple-breath washout (MBW) is a lung function test that measures this inhomogeneity in the lung. However, the factors that influence the results of the test and differentiate between health and disease are not well understood. Computational models help us to understand better the relation between anatomical structure and physiological properties of the lung, but none of them has dealt in depth with the MBW test in whole. Our aim was to create a lung model that simulates the entire MBW test and study the role of lung structure and tissue mechanics on the washout procedure. We developed a multi-scale lung model to simulate the inert gas transport in all airways including the gas exchange area. Our model offers the opportunity to understand the ventilation distribution in the healthy lung. It can also mimic certain patterns of lung disease by applying modifications in mechanical properties out of the physiological limits. Thus, it can be used to study MBW characteristics in health and disease.

## Introduction

The multiple-breath washout (MBW) is a lung function test that measures the degree of ventilation inhomogeneity [1] and is increasingly used for both research and clinical purposes in patients with obstructive lung disease, such as cystic fibrosis, primary ciliary dyskinesia, etc. [2–4]. The test is based on the clearance of a tracer gas during multiple tidal breaths. Each MBW test comprises a washin and a washout phase. During the washin phase, the tracer gas (normally an inert extrinsic gas) is delivered in a known concentration. When the tracer gas concentration reaches an equilibrium in the lung, the washout phase starts. In the case of inert intrinsic gases (N_2_), the test is simplified, as no washin phase is needed. In the washout phase the subject inhales a gas other than the tracer gas (e.g. pure O_2_ in case of N_2_MBW), so that the lungs wash out the tracer gas gradually by each expiration [2]. The progressive decrease in tracer gas concentration during the washout (washout envelope) as well as the breath-by-breath analysis provide useful information about the distribution of ventilation within the lung [1], and for this reason the test is of increasing importance for the medical community. However, the biomechanical phenomena that influence the specific washout profile of a tracer gas are not well understood.

The anatomical diversity in the airways as well as the physiological properties of the lung tissue (e.g. compliance, resistance) influence the respiratory function in a complex way [5, 6]. Over the last years, computational models have been used to better understand those interactions. Anatomically based three-dimensional (3D) lung models have elegantly simulated lung tissue mechanics [7–9] and 3D fluid dynamics in large airways [10–12], but without specific focus on the ventilation and gas transport in the entire lung in the context of MBW. More simplified models have addressed these phenomena, but either have not included physiological asymmetries in lung morphology, or have not modeled the whole lung [13–23]. Therefore, results of these models cannot be directly compared to clinical data in humans [24].

The aim of this article is to introduce a computational model that simulates gas transport in the entire lung during the MBW test, taking into account transport phenomena at different scales. It should allow relating physiological phenomena at the smallest scales of the lung to gas concentrations at the mouth, which can be measured clinically. Such simulations can provide detailed insight in the gas transport dynamics during the MBW in different airways and help to understand better the effect of lung morphology and tissue mechanics on tracer gas washout. To this end, structural data from human lungs have been used to construct a fractal lung model, taking into account morphological and physiological asymmetries in lung anatomy [15, 25, 26] including the number and size distribution of the acini [27, 28]. We have designed the model in a way that is complex enough for the introduction of different types of asymmetries and structural properties of the acini, but simple enough to allow for quick computational turn-around times on desktop computers. The model was used to study some basic mechanisms, which lead to well-known clinical observations in MBW [1], and an example of a model for the healthy lungs was created, using known parameters that can produce a physiological heterogeneous ventilation distribution, as described in healthy individuals [6].

Such models inherently lack suitable validation methods [29] because ground truth data is not available in the lower airways. Here, we compared the MBW simulations in the computational lung model to data from nitrogen MBW (N_2_MBW) tests from healthy controls (N = 4). Although this comparison is too small to serve as a statistically solid validation of the model, the good agreement between computational results and in vivo data illustrates the potential of the proposed model to reproduce clinical test data.

## Methods

### Ethics statement

The study was approved by the Ethics Committee of the Canton of Bern, Switzerland (KEK-Gesuchs-Nr: 181/03), and caregivers gave written informed consent.

### Lung morphology

In our model, the airway morphology is represented by a generic dichotomous tree network of straight branching pipes [30], which terminates in trumpet-like compartments. The straight pipes represent the larger, non-compliant airways, where convective transport is dominant. The dimensions of the trachea and the relative dimensions (i.e. with respect to the trachea) of the airways in the first four generations are defined according to anatomical data from Weibel [26]. In addition, the entire airway network of pipes (all conducting airways including the trachea, without the trumpet-like compartments) was then scaled to meet a specific functional residual capacity (FRC) that is, the air volume that remains in the lung after tidal expiration. The length of the scaled trachea was defined as

$$l_{0}={l_{0}}_{W}{\left(\frac{FRC}{FRC_{W}}\right)}^{1/3}$$ (Eq 1)

where the subscript W indicates a reference quantity [26].

For airways past the 4^th^ generation, we applied the scheme for a regular branching asymmetry introduced by Majumdar et al. [25, 30]. In this scheme, each pipe-like airway bifurcates in a major and a minor daughter airway. In the airway network every parent, minor, and major daughter share a common node. The dimensions of the daughter airways, namely their diameter and length, are different fractions of the dimensions of their common parent pipe,

$$\begin{array}{l} d_{z+1_{maj}}=d_{z}\kappa_{maj}\;\mathrm{with}\;\kappa_{maj}={\left(1-r\right)}^{1/\eta }\;\mathrm{and}\\ d_{z+1_{min}}=d_{z}\kappa_{min}\;\mathrm{with}\;\kappa_{\min}=r^{1/\eta } \end{array}$$ (Eq 2)

where d_z_ is the diameter of a pipe at generation z, and r and η denote the asymmetry parameter and reduction rate, respectively. The same scheme was used to define the airway lengths. Majumdar et al. proposed values *r* = 0.326 and *η* = 2.97 for which the resulting structure best represents the human lung on a statistical basis [25]. In the present model, this bifurcation scheme was applied recursively until the diameter of a pipe was less than a lower limit diameter *d*_*lim*_ = 1.8 *mm*. This limit defines the terminal pipes in the model and was chosen to represent bronchi at the 8^th^-12^th^ generation. The generation of the individual terminal pipe-like airways depends on the limit diameter and varies within the model, due to the asymmetric bifurcation scheme. This limit diameter was chosen in order to complete the simulations with reasonable computational costs. A numerical experiment showed very small differences in the simulation outcomes for *d*_*lim*_ = 1.6,1.8,2.0 *mm*.

**Fig 1:**
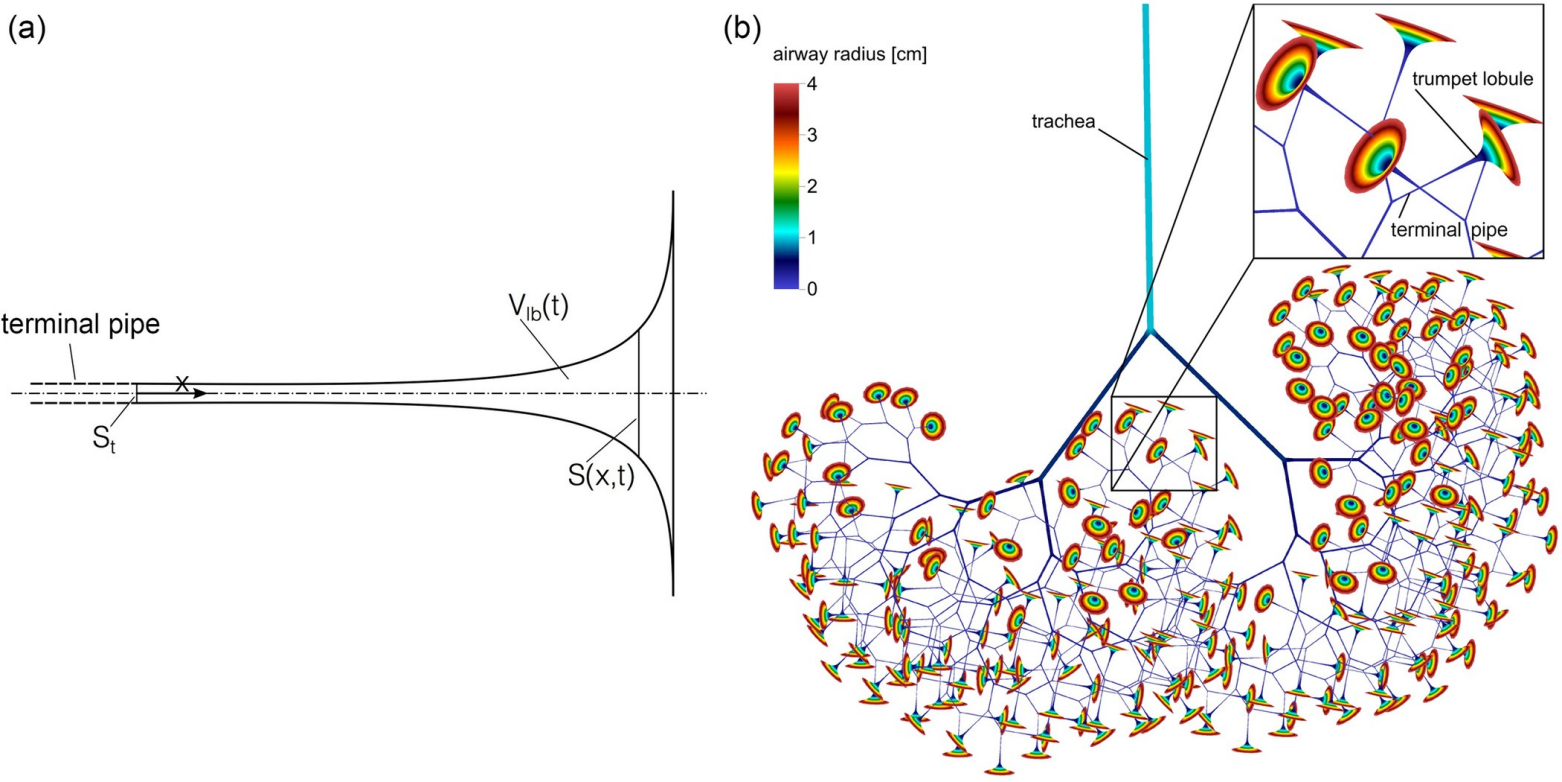
Trumpet lobule geometry and lung model morphology. (a) Schematic representation of the trumpet model used for the lobules with the cross-section of the terminal pipe S_t_ and the time-variable cross-section S(x,t) and volume V_lb_(,t) of a trumpet lobule model. (b) Idealized lung model morphology based on bifurcation rule (Eq 2). For better visibility of the airway structure, all diameters were scaled with the factor 0.1. The length and diameter ratio of airways until the fourth generation are irregular and follow from empirical data by Weibel [26]. After the fourth generation, airways bifurcate in a minor and major daughter airway according to Eq 2. From this scheme an asymmetric network of airways with different total lengths results. With each generation, airways get smaller, both in terms of length and of cross-section. In the lung model, when the diameter of the airways falls below a limit diameter d_lim_, a so-called terminal pipe is reached, and different model (the trumpet lobule) is used to represent smaller airways.

The computational unit distal to a terminal pipe constitutes a lobule and was modelled using a trumpet-like compartment (trumpet lobule). Please note that the “lobule” as defined here differs from the anatomical term lobule as defined by Miller [31], because it contains, apart from the acini that include respiratory bronchioles, alveolar ducts and alveoli, also the generations of conducting airways with diameter smaller than d_lim_. Unlike the pipes, the trumpet lobule is a compartment with diverging, time-variable cross-section (Fig 1A). The number of these trumpet lobules is equal to the number of terminal pipes, as each terminal pipe leads to one lobule.

The residual volume of the trumpet lobules was defined such that the total volume of the model (pipes and trumpet lobules) at the end of a tidal expiration equaled a predefined FRC. Fig 1B shows a sketch of a model lung illustrating the relative scales of a network of airways defined by the bifurcation rule (Eq 2).

### Mathematical description of the trumpet lobule

For the trumpet model representing the lobules and their peripheral airways, a model for the total cross-section of the trumpet lobule S_lb_(x,t), as well as for the mean advection velocity u_lb_(x,t) had to be derived.

The flow rate at the inlet of each trumpet equals the flow rate in a terminal pipe and Q_t_(t) was known from the results of a model for lung ventilation (described in the upcoming section) and was used to compute the total volume of the trumpet $V_{lb}(t)=\int_{0}^{t}Q_{t}dt+{V_{lb}}^{0}$.

The initial volume of the trumpet lobule, *V*_*lb*_(*t* = 0) = *V*_*lb*_^0^, followed from FRC-based scaling of the lung model.

Assuming a uniform homothety ratio of *κ* = 0.85 [19] for airways lumped in a trumpet lobule, the total change of cross-section along the streamwise coordinate x can be described as

$$S(x,0)=S_{t}{\hat{\kappa }}^{z(x)},\;\mathrm{with}\;\hat{\kappa }=2\kappa^{2}$$ (Eq 3)

where S_t_ is the cross-section of the terminal pipe, and z(x) is the generation at position x with respect to the inlet of the trumpet lobule where z = x = 0.

Considering l_t_ to be the length of the terminal pipe, the cumulative length at generation z (with respect to the inlet of the lobule) would be $\sum_{k=1}^{z}l_{t}\kappa^{k}$.

The limits of this sum for *z*→∞ are

$$\underset{z\to \infty }{lim}\sum_{k=1}^{z}l_{t}\kappa^{k}=l_{t}\kappa /(\kappa -1))≕L.$$ (Eq 4)

From these relations, an expression for the generation in function of the distance to the inlet of the trumpet can be computed,

$$z(x)=\frac{log[x\frac{\kappa -1}{\kappa l_{t}}+1]}{log(\kappa )}$$ (Eq 5)

Using Eq 3 together with Eq 4 for further treatment of the lobule model becomes a rather cumbersome task. We therefore sought a model S_lb_(x,t), which approximates Eq 5 but allows to derive an analytical expression for u_lb_(x,t).

To this end, we used a power law of the form

$$S_{lb}(x,t)=p_{1}x^{n_{1}}+p_{2}x^{n_{2}}+S_{t},$$ (Eq 6)

with n_1_, n_2_ = 20, 2, where the coefficients p_1_ and p_2_ were defined such that the lobule cross-section S_lb_ intersects with Eq 3 at a chosen generation z*, and the prescribed initial volume V_lb_^0^ of the trumpet lobule is obtained for a given length l_lb_ of the trumpet lobule. The mathematical expression for this parameter definition as well as a graphic comparison between the formulation Eq 3 and the model Eq 6 are provided in S1 Appendix (Section 1).

The expression given in Eq 6 determines the shape of the trumpet lobule at any time.

An important feature of this model is the major contribution of peripheral airway (where x is close to l_lb_) to the overall expansion of the lung. Furthermore, the differentiation with respect to time of Eq 6 as well as the integration along the trumpet centre line coordinate x is straightforward and therefore allows to formulate a modified advection diffusion equation for the trumpet lobule (see S1 Appendix, Section 1)

Apart from the constraint on the total volume, the geometrical and mechanical properties of the stiff (pipes) and the compliant parts (trumpet lobules) can be modified individually to study systematically the effects of structural lung inhomogeneity.

### Ventilation

**Fig 2:**
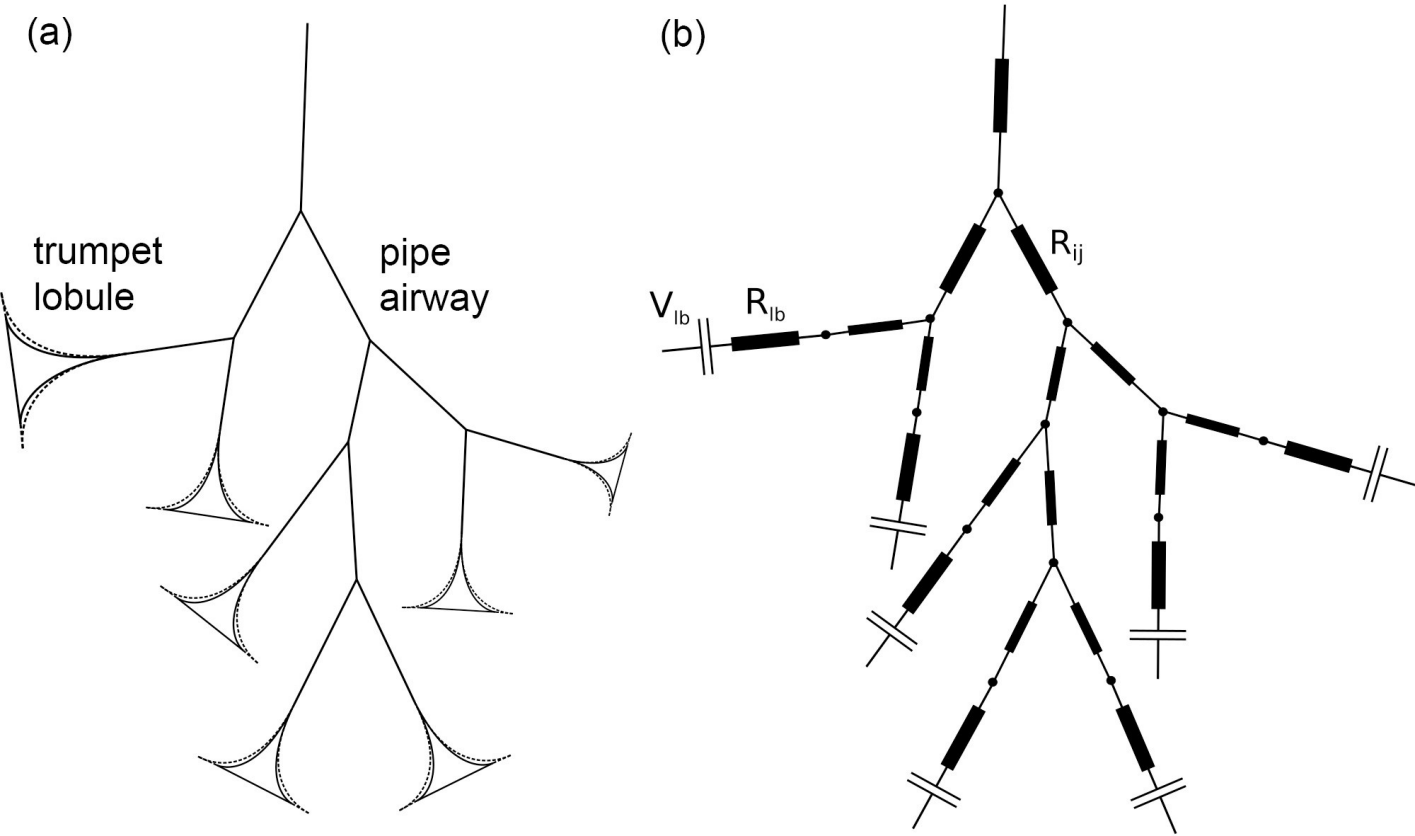
Ventilation lumped parameter model schematics. (a) Network of pipe- and trumpet-like elements used for the upper and lower airways, respectively. (b) Corresponding lumped parameter model composed of resistances for the conducting airways R_ij_ and for the trumpet lobule R_lb_ and compliance elements which relate the trans-lobular pressure to the volume of the trumpet lobule V_lb_(t) (see Eq 7).

Lung ventilation was simulated by a lumped parameter (0-dimensional) model (Fig 2) based on the model morphology described above. For the pipe-like airways, purely resistive elements were used. *Womersley*'s theory for pulsatile flow in tubes [32] was applied to account for inertial effects during normal breathing, which have a considerable effect on the flow resistance in bigger airways until the fifth generation [33] (see also S1 Appendix). In general, the pressure difference between two subsequent nodes with index i and j in the network of pipe-like airways reads *p*_*i*_−*p*_*j*_ = *R*_*ij*_*Q*_*ij*_. Here Q_ij_ is the flow rate from node i to node j, and the hydrodynamic resistance R_ij_ depends on the radius r_ij_ and the length l_ij_ of the conducting airway between two nodes, and on the breath period T_B_. More information on the pressure-flow relation can be found in S1 Appendix.

**Fig 3:**
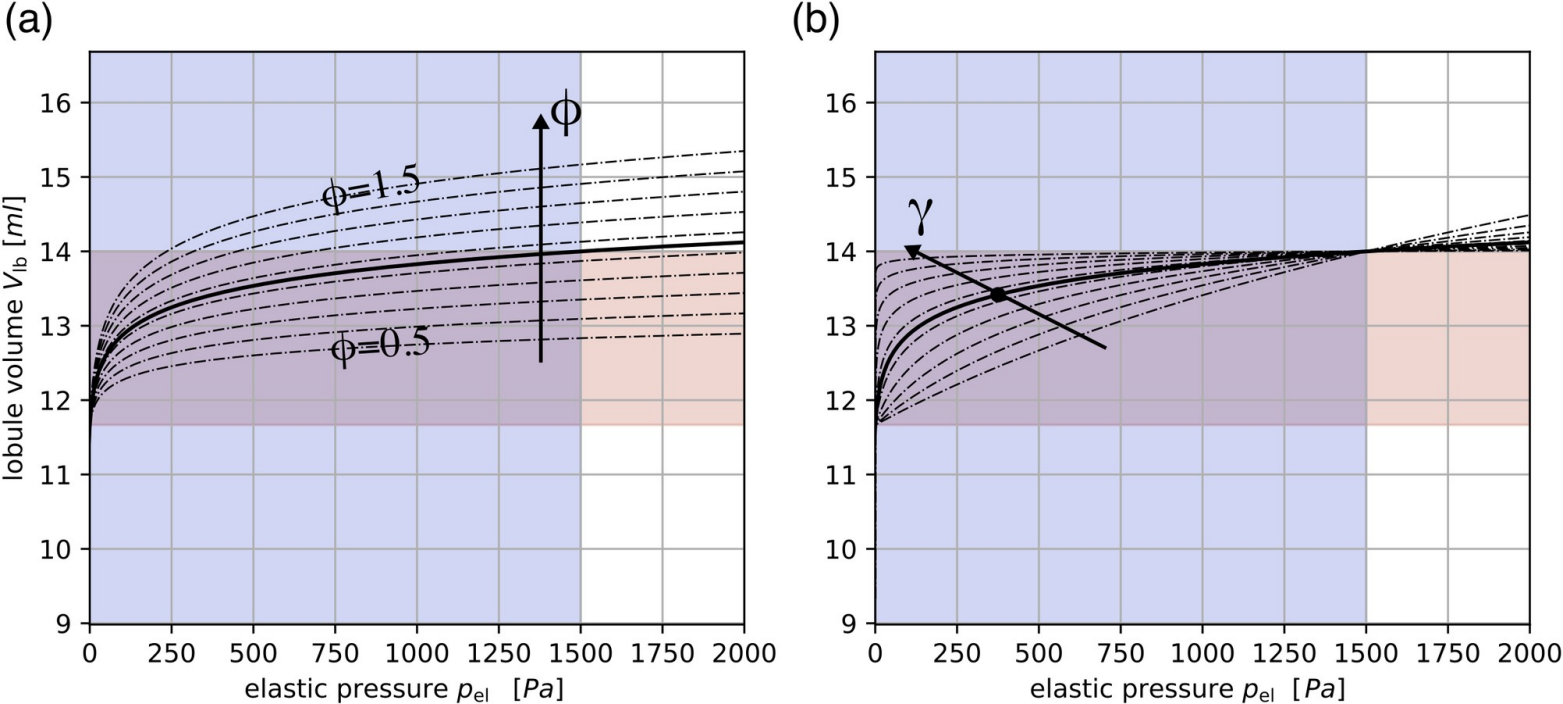
Pressure-volume curve for trumpet lobule. Lobular volume V_lb_ in function of the elastic pressure p_el_ as defined in Eq 3. The purple area indicates the reference pleural pressure range $p_{lb}^{TV}$ and the orange area indicates the nominal lobule volume range $\left[V_{lb}^{0},V_{lb}^{0}+V_{lb}^{TV}\right]$. In panel (a) the influence of the compliance modification parameter ϕ∈[0.5,1.5] is shown. For lobules with normal compliance (ϕ = 1, solid line) the elastic pressure curve intersects in the point $\left(p_{lb}^{TV},V_{lb}^{0}+V_{lb}^{TV}\right)$. In panel (b) the effect of the non-linear parameter γ is shown. The parameter was chosen such that the elastic pressure curve intersects with the point $\left(\frac{1}{4}p_{lb}^{TV},V_{lb}^{0}+\frac{3}{4}\phi V_{lb}^{TV}\right)$ (black dot). Note that ϕ = 1 was used for all graphs in panel (b).

The trumpet lobule model, mainly representing compliant airways, is composed of a nonlinear compliance element (elastic pressure, p_el_) and a resistance element (viscous pressure loss, p_diss_), acting in series between a node i, corresponding to a terminal pipe, and the pleural gap with pressure p_pl_. This representation is based on the pressure-volume relation as presented by Bates [34]. However, instead of the regular linear elastic law, we used an empirical exponential expression to represent p_el_. The corresponding pressure difference was defined as

$$p_{i}-p_{pl}=\underset{p_{el}}{\underbrace{\beta e^{\gamma V_{lb}^{0}}(e^{\gamma {\tilde{V}}_{lb}}-1)}}+\underset{p_{diss}}{\underbrace{R_{lb}Q_{lb}}}$$ (Eq 7)

where R_lb_, $V_{lb}(t)=V_{lb}^{0}+{\tilde{V}}_{lb}(t)$ and Q_lb_ denote the total flow resistance of the lobule, its volume and the flow rate into the trumpet lobule, respectively; $V_{lb}^{0}$ is the lobule volume at FRC and ${\tilde{V}}_{lb}(t)$ is the dynamic volume during breathing [34]. The shape parameters β and γ were used to define and modify the degree of the compliance parameter φ (i.e. the distensibility of the lobule, described below in details) and the non-linearity (in the pressure-volume relation) of the trumpet lobule, respectively. There is a single β-γ pair for each lobule, and both are defined indirectly by fixed intersection points in the pressure-volume curve for the elastic pressure (see Fig 3A). More information on the mechanical properties of the model can be found in the Section “Structural and mechanical modifications of the model”.

To ensure mass conservation, the flow rates in and out of each node i satisfy the balance ∑*Q* = 0. At the inlet, i.e. upstream node of the trachea, the flow rate is defined by the predefined boundary conditions *Q*_*in*_(*t*), and for the trumpet lobule the flow rate of the terminal pipe Q_t_ equals the change of volume of the lobule,

$$Q_{t}=\frac{dV_{lb}}{dt}.$$ (Eq 8)

This results in a system of differential algebraic equations (DAE). Together with appropriate boundary conditions, the DAE system governs the flow distribution in the lumped parameter model. A detailed description of the numerical implementation of the lumped parameter model is provided in S1 Appendix.

During inspiration, gas is transported from the mouth and the nose toward the alveolar membrane, as a result of the motion of the diaphragm and the thoracic cavity, causing a volume increase in the lung and a pressure decrease in the pleural gap. This negative (relative) pressure in the pleural gap causes a pressure gradient across the peripheral lung tissue and along the airways to the mouth and nose. Therefore, the pleural pressure and the pressure at the mouth would be obvious choices for the boundary conditions for the lumped parameter model (LPM). However, the pleural pressure is in general not measurable during clinical routine. Instead, the pleural pressure was determined computationally such that a prescribed flow rate *Q*_*in*_(*t*) was attained in the trachea. This flow rate could be easily determined during clinical testing.

### Gas transport

Gas transport in the lung occurs by convection and diffusion [35, 36]. To model the distribution of MBW tracer gases in the lung, typically N_2_ or sulfur-hexafluoride [1], a one-dimensional advection-diffusion transport equation for a scalar variable representing the normalized gas concentration *c* = *c*(*x*,*t*)∈[0,1] was solved with a finite difference method (detailed description in S1 Appendix). Simulation of MBW involved the computation of gas concentration in all model airways over multiple breath periods. We considered only inert gases for which the dominant transport mechanisms are advection by the carrier gas (ambient air) and diffusion. The transport process was modelled along the centerlines of each pipe-like airway and along the axis of each lobule trumpet. The 1D approximation entails consideration of averaged (lumped) quantities within a cross-section, or in case of the trumpet model, within all airways represented by the cross-section of the trumpet at a given axial position. Advection-diffusion processes in a pipe-like geometry are subject to high radial velocity gradients, which can lead to radial concentration gradients such that the average effective diffusivity within a cross-section increases. In the human lung, these phenomena can take place at different scales: In the upper airway, where the *Reynolds* number *Re*_*d*_ (based on the airway diameter d) is about 10'000, turbulent flow strongly enhances mixing. In smaller airways (*Re*≪2000), *Poiseuille* flow with a parabolic velocity profile can be assumed. Enhanced diffusion due to high velocity gradients was modelled based on the concept of *Taylor dispersion*, where the local diffusion coefficient is a function of the local *Peclet* number [37],

$$\hat{D}=D\left(1+\frac{1}{192}{Pe}^{2}\right)$$ (Eq 9)

where $\hat{D}$ and D are the effective and molecular diffusion coefficients, respectively, and *Pe* = *ud*/*D* is the *Peclet* number defined with the local mean velocity u and the local airway diameter d. This is an approximate measure. Taylor dispersion is a concept, which strictly applies only to developed flow in straight tubes. Hence, the modification of the diffusion coefficient (Eq 9) does not account for local changes in diffusivity due to secondary flow phenomena occurring at bifurcations and curved airways, and likely underestimates the level of diffusion enhancement.

The advection-diffusion equation for gas transport was solved separately in each airway, applying interface conditions at the bifurcation nodes to couple the transport between different airways. We considered a transport equation of the following general form

$$\frac{\partial (Sc)}{\partial t}+\frac{\partial F}{\partial x}=0$$ (Eq 10)

with the flux

$$F=S_{ad}uc-S\hat{D}\frac{\partial c}{\partial x}$$ (Eq 11)

Here, we distinguished between the total cross-section S and the cross-section S_ad_ in which advection with the carrier gas velocity u(x,t) takes place. This assumption is important, because the airway geometry becomes increasingly complex (i.e. non-tubular, alveolar ducts and alveolar trees) for smaller airways, and tracer gas advection does not occur in the entire lumen [35, 38, 39]. At bifurcations, the concentration flux was conserved by enforcing ∑*F* = 0. In S1 Appendix, a model for the trumpet lobule is derived which allows a more specific form of Eq 10 to be stated for the gas transport within trumpet lobules with non-constant cross-section. In addition, the finite difference scheme for spatial derivatives and time-integration method used for the numerical solution of Eq 10 are explained.

### Structural and mechanical modifications of the model

The proposed multi-scale model was designed to study the effects of functional and structural inhomogeneities of the peripheral airways on the N_2_ washout procedure. To illustrate the capabilities of the model, several parameters of the trumpet lobules were systematically modified. The parameters were defined in a way that their effect on lung geometry and mechanics was physically meaningful and intuitively clear:

1. Lobule Compliance: Lung compliance, as a measure of distensibility of the lung, is not uniform within the healthy lung in vivo [40]. Moreover, it is found impaired in many lung diseases, e.g. increased in emphysema, and decreased in pulmonary fibrosis [41–43]. We tuned tissue compliance with the parameter *ϕ*, which is a non-dimensional factor that has a stiffening or softening effect on the elastic pressure. In the following the meaning of *ϕ* is explained in more detail: We considered a mean tidal volume $V_{lb}^{TV}=TV/N_{lb}$ per lobule, where TV is the tidal volume, and *N*_*lb*_ is the total number of trumpet lobules. We then defined the nominal lobule volume range $\left[V_{lb}^{0},V_{lb}^{0}+V_{lb}^{TV}\right]$. Furthermore, we considered a reference pressure amplitude $p_{lb}^{TV}=1500$ Pa, which corresponds to a typical pressure range due to pleural pressure changes [44, 45]. For a given pressure load $p_{lb}^{TV}$, the elastic component of the lobule mechanics can be considered stiff (reduced compliance) if its maximum dynamic volume is smaller than $V_{lb}^{TV}$ (*ϕ*<1), and soft (increased compliance, *ϕ*>1) otherwise. Fig 3A shows the elastic pressure curve (Eq 7) for a lobule for different values of *ϕ*, which effectively modify the curve via the shape parameter β as
  $$\beta =\frac{p_{lb}^{TV}}{e^{\gamma V_{lb}^{0}}(e^{\phi \gamma V_{lb-1}^{TV}})}$$ (Eq 12)
  The remaining shape parameter γ accounts for the non-linearity of the elastic pressure curve (Fig 3B). It was computed numerically for all the lobules (using a minimization approach) such that the elastic pressure curve intersects with the point $\left(\frac{1}{4}p_{lb}^{TV},V_{lb}^{0}+\frac{3}{4}\phi V_{lb}^{TV}\right)$ in the volume-pressure plane. This point was chosen because the resulting curve qualitatively lies between a linear (*γ*→0) and an almost right-angled curve (*γ*→∞).
2. Lobule Volume: Volume differences between the respiratory units (i.e. the anatomical lobules) but also within each unit have been described in healthy lungs [6, 46] and are more pronounced in obstructive lung disease [47]. We used the modification parameter *θ* to modify the residual volume of a trumpet lobule as $V_{lb,mod}^{0}=\theta V_{lb}^{0}$, such that *θ*<1 resulted in reduced residual trumpet lobule size.
3. Lobule Resistance: Differences in airway resistance [34] between separate airspaces are known to affect the washout process [48]. Such differences were introduced via a modification of the pressure loss (see Eq 7) in the trumpet lobule *p*_*diss*,*mod*_ = *τR*_*lb*_*Q*_*lb*_, where the modification parameter *τ*>1 directly modifies the lobular flow resistance R_lb_. This can be used to model the obstruction of airways in a lobule, e.g. due to mucus plugging.

### Comparison of model simulations with in vivo MBW measurements in healthy children

The aim of the comparison of model results with in vivo data was to demonstrate that the model can relate microscale modifications at the lobular level to clinically observed MBW metrics (MBW washout envelope and phase III slope analysis [1]).

We used data from healthy adolescents recruited for lung function studies in the Inselspital Children’s University Hospital, Bern, Switzerland. N_2_MBW measurements (N = 4) were collected according to the recent ERS/ATS consensus guidelines [1] using the ultrasonic flowmeter (Exhalyzer D, Eco Medics AG, Duernten, Switzerland) and the software provided by the manufacturer (Spiroware 3.1.6) as previously described [49]. During the test, the subject sat in an upright position wearing a nose-clip and was asked to breathe regularly through a snorkel-like mouthpiece connected to a bacterial filter and a dead space reducer.

## Results & discussion

We use the above described model of the whole lung to study the effects of structural and mechanical modifications on MBW outcomes.

### Baseline configuration vs. modified lobular compliance

**Fig 4:**
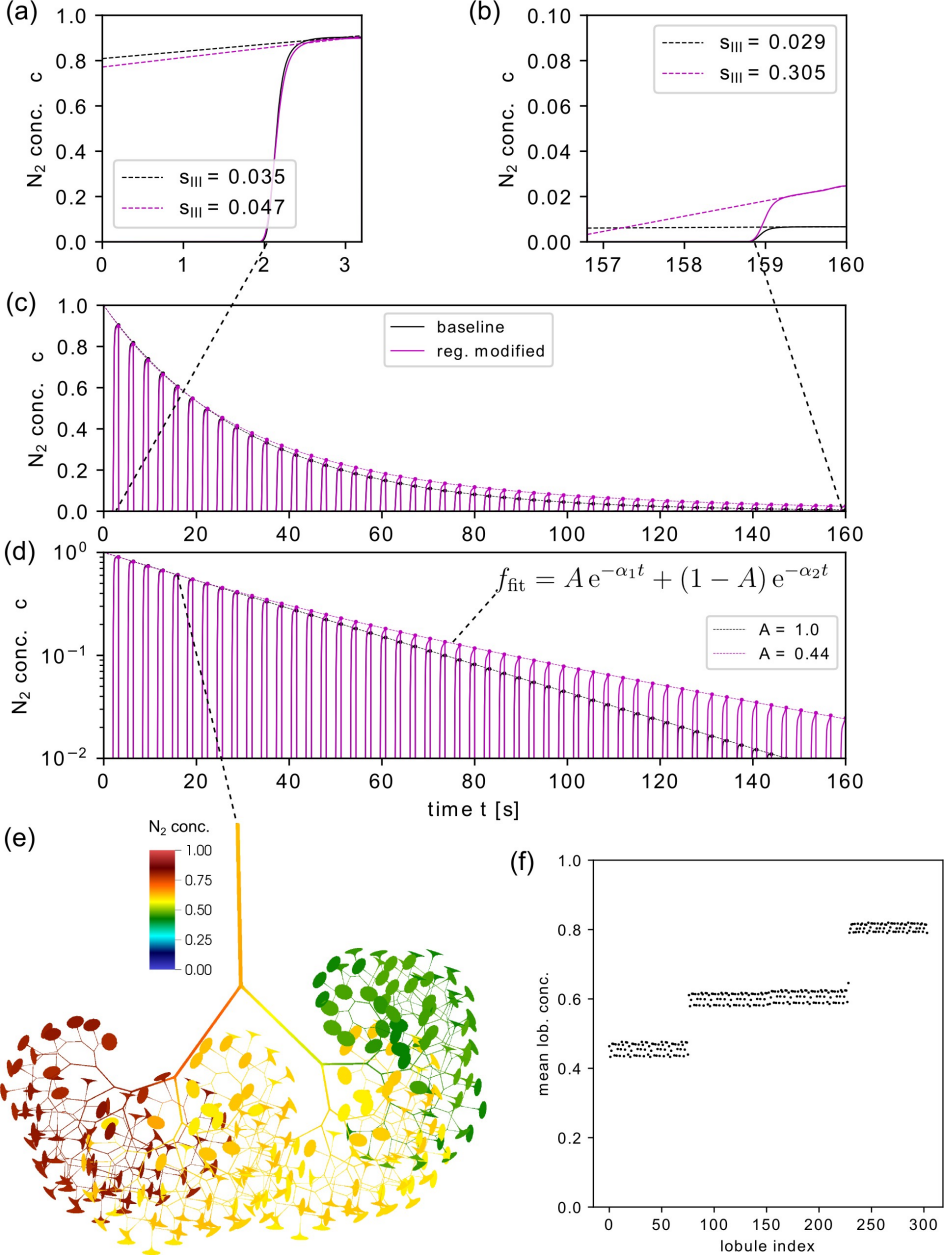
Overview of different simulation results for baseline model configuration and modified compliance. Results of the simulated N_2_ gas washout for the baseline configuration (uniform and constant lung model parameter, black) and for altered trumpet lobule compliance using ϕ = 0.5, 1.5 in two different regions, respectively, each accounting for 25% of all trumpet lobules (purple color). Normalized phase III slopes s_III_ are shown for the first (a) and last (b) breath. The washout profile (N_2_ concentration at the entrance of the trachea) in (c) linear scale and (d) logarithmic scale for 50 simulated breaths with a uniform concentration decay for the baseline model, and a non-uniform decay for the regionally modified model (slow-fast washout profile). Panels (e) and (f) illustrate further the model with modified lobular compliance at the end of the fifth breath: (e) spatial N_2_ concentration distribution where the trumpet lobules parametrized with lower and higher compliance are located on the left and right side of the airway tree, respectively. In (f) the mean lobular N_2_ concentration is shown for each trumpet lobule.

**Fig 5:**
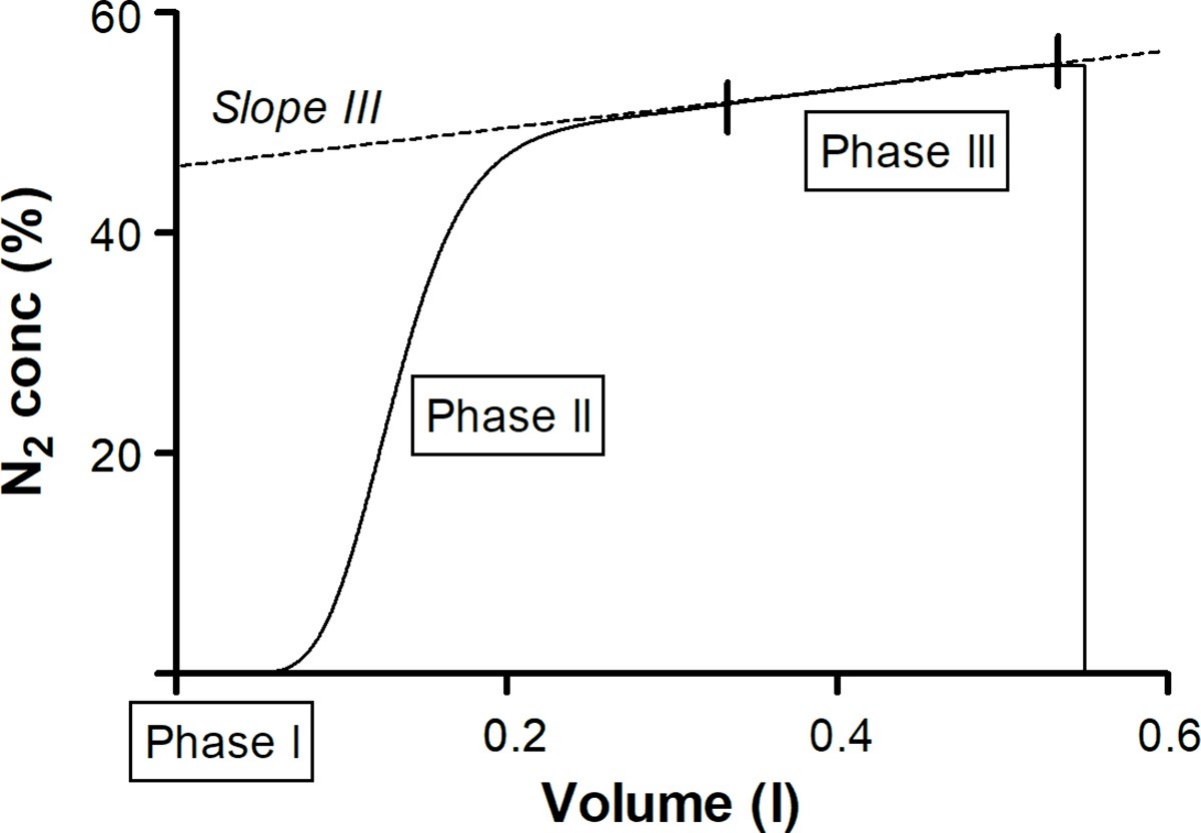
Schematic presentation of the N_2_ concentration-volume curve of a single breath. Expired N_2_ concentration is expressed as % of the initial N_2_ concentration. Phase III is defined between 50% and 95% of the expired volume. Slope III: the slope of the concentration-volume curve during phase III.

A baseline simulation has been performed with the parameter settings listed in Table 1, but without any added asymmetries with respect to lobular compliance, lobular residual size, or lobular resistance. We compared this reference simulation with results for modified lobular compliance where two regions (each accounting for 25% of all trumpet lobules) were modified using *ϕ* = 0.5 and *ϕ* = 1.5, respectively. Fig 4 shows the results for both simulations (baseline and compliance modifications). The washout curve (Fig 4C and 4D) can be analyzed in different ways: Each expiration starts with zero N_2_ concentration (phase I), which corresponds to the washout of the dead space (the pipes in the model), and then a rise in N_2_ concentration, which is first very quick (phase II) and later slow (phase III) [50–52]. Phase III is the part of the concentration-expired volume curve that corresponds to 50% to 95% of the expired volume per breath [1] (Fig 5). The end-expiratory N_2_ concentration diminishes from one breath to the next as the N_2_ washout progresses. The ratio of two subsequent end-expiratory N_2_ concentration values, i.e. the decay of the washout curve envelope, provides information about the gas washout efficiency of the whole lung, and indirectly about the ventilation of lung compartments. For the baseline model configuration, this exponential decay is uniform (linear envelope graph in a semi-logarithmic plot over time, in Fig 4D). In the model with regions of variable lobular compliance, the decay is non-uniform and delays towards the end. This can be associated with a non-uniform ventilation in different regions of the lung. To quantify these washout properties, an exponential function of the following form was fitted to the envelope of end-expiratory values.

$$f(t)=Ae^{-\alpha_{1}t}+(1-A)e^{-\alpha_{2}t}$$ (Eq 13)

Here, *α*_1_ and *α*_2_ (with *α*_1_,*α*_2_>0) are the decay rates of two superimposed processes, one fast and one slow [53], and *A*∈[0,1] represents the relative weight of the slow decay process (*α*_1_<*α*_2_). The decay of the process is considered uniform if *A* = 1 and non-uniform if *A*<1. Changing the compliance properties as described above resulted in *A* = 0.44, *α*_1_ = 0.018, and *α*_2_ = 0.045, compared to *A* = 1.0 and *α*_1_ = 0.031 for the baseline.

**Table 1:** Parameter settings of the baseline model.

Breathing profile	Tidal volume	TV	[l]	0.5
	Breath period	T_B_	[s]	3.2
	Inlet flow profile			Sine function
	Length of simulation			50 breaths
Model settings	Functional residual capacity	FRC	[l]	3.0
	Limit diameter pipe airways	d_lim	[mm]	1.8
	Number of lobules		[–]	304
	Pipe generations			7–11*

* The number of pipe generations is not uniform for all model lobules, due to the asymmetrical branching scheme.

The spatial distribution within the lung model after the fifth breath (Fig 4E and 4F) provides further insight to the non-uniform (i.e. heterogeneous) washout process. The concentration remains high in lobules with decreased compliance (*ϕ* = 0.5) and is reduced more rapidly in those with increased compliance (*ϕ* = 1.5). Later in the washout process, the relative concentration difference between the two regions is therefore higher than in the baseline configuration and the washout slows down due to the increasingly dominant contributions of the slowly washed-out units.

The concentration profile can be further analyzed and interpreted on a per-breath basis. The slope of the concentration-volume curve during phase III (*slope III*) indicates whether the gas mixtures from different lung regions have different gas concentrations [35, 50] (Fig 5). Therefore, *slope III* has been linked to inhomogeneous gas transport dynamics, and parameters derived from this analysis are of increasing clinical relevance [54–56]. To quantify the phase III profile in our study, the parameter *s*_*III*_ is defined, which is the slope of a linear function fitted to the N_2_ concentration values corresponding to the phase III, normalized with the mean N_2_ concentration during phase III (Fig 5):

$$S_{III}=\frac{Slope\;III}{{mean\;N}_{2}\;(phase\;III)}$$ (Eq 14)

Comparing the baseline washout with the washout of modified compliance, *s*_*III*_ was higher (steeper slope) in the modified lung already for the first breath (Fig 4A), and this difference in *s*_*III*_ increased further until the last breath (Fig 4B).

### Outcome parameters for comparison

For the comparison of other types of lung model modifications that represent a specific kind of structural and functional lung asymmetry, we used the weight parameter *A* and the decay rate *α*_1_ (and *α*_2_) from the fitting function (Eq 9) approximating the washout envelope, as well as the clinically important parameter *s*_*III*_ (Eq 14).

### Other types of regional lobular modifications

In separate simulations, the residual volume of trumpet lobules was altered using *θ* = 0.5 for a subset of lobules accounting for 25% of all trumpet lobules. Note that the model is designed such that it scales the residual volume of the remaining 75% of lobules automatically to preserve the prescribed FRC of the lung. In another simulation, the resistance was increased (*τ* = 8) in 25% of the lobules, which corresponds to a local reduction of airway diameters by approximately 40%.

**Fig 6:**
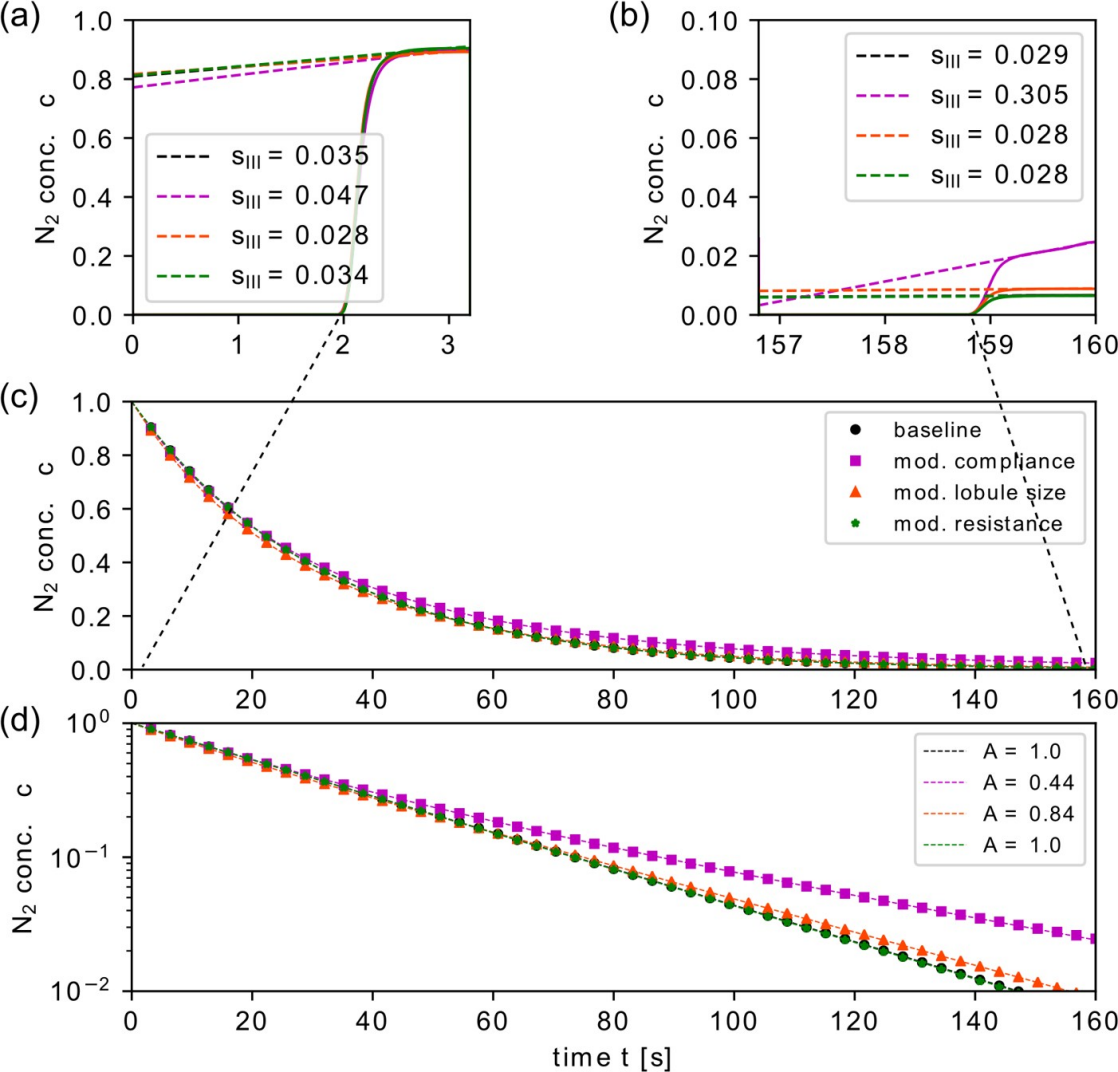
N_2_ washout for baseline model configuration and modified trumpet lobule properties. Results of the simulated N_2_MBW for the baseline configuration (uniform and constant lung model parameters) and for three cases where different trumpet lobule parameters were modified regionally: 1) Lobular compliance was altered using ϕ = 0.5,1.5 in two regions (same as for results shown in Fig 4), 2) the lobular residual volume was decreased using θ = 0.5, 3) the hydrodynamic resistance was amplified by a factor of 8 for 25% of all lobules. Normalized phase III slopes s_III_ are shown for the first (a) and last (b) breath. (c, d) The washout (only the envelope of the N_2_ end-expiratory concentrations (symbols) and the corresponding fitting functions (dashed line, Eq 13) are shown) for 50 simulated breaths.

Fig 6 shows the simulation results for these three modified cases together with the baseline configuration. Note that the baseline results (black line in Fig 6) are barely visible, because some of the simulations in the model with increased resistance yielded very similar results. The compliance modifications showed the strongest effect and yielded a non-uniform, delayed washout (Fig 6C and 6D). While the resistance modification did not lead to any notable difference from the baseline result (*A* = 1.0), heterogeneous residual lobular volume also yielded a non-uniform washout (*A* = 0.84) similar but weaker than the model with modified compliance. Interestingly, the washout was delayed in later breaths in the case of modified residual lobular volume, and of compliance modifications. The modified residual volume caused in addition a faster decay for early breaths (Fig 6C before *t* = 60 s). This was also reflected in the slow (*α*_1_ = 0.028) and fast (*α*_2_ = 0.074) decay rates, e.g. when compared to the decay rate for the baseline configuration (*α*_1_ = 0.031). A reason for this could be that in smaller and shorter lobules, the flow of pure oxygen replaces a larger fraction of N_2_ and thus the washout per breath is more efficient (faster). Although the residual lobule size is different, a similar flow rate of pure oxygen is preserved in all lobules as long as the compliance properties and the pleural pressure distribution remain uniform.

**Fig 7:**
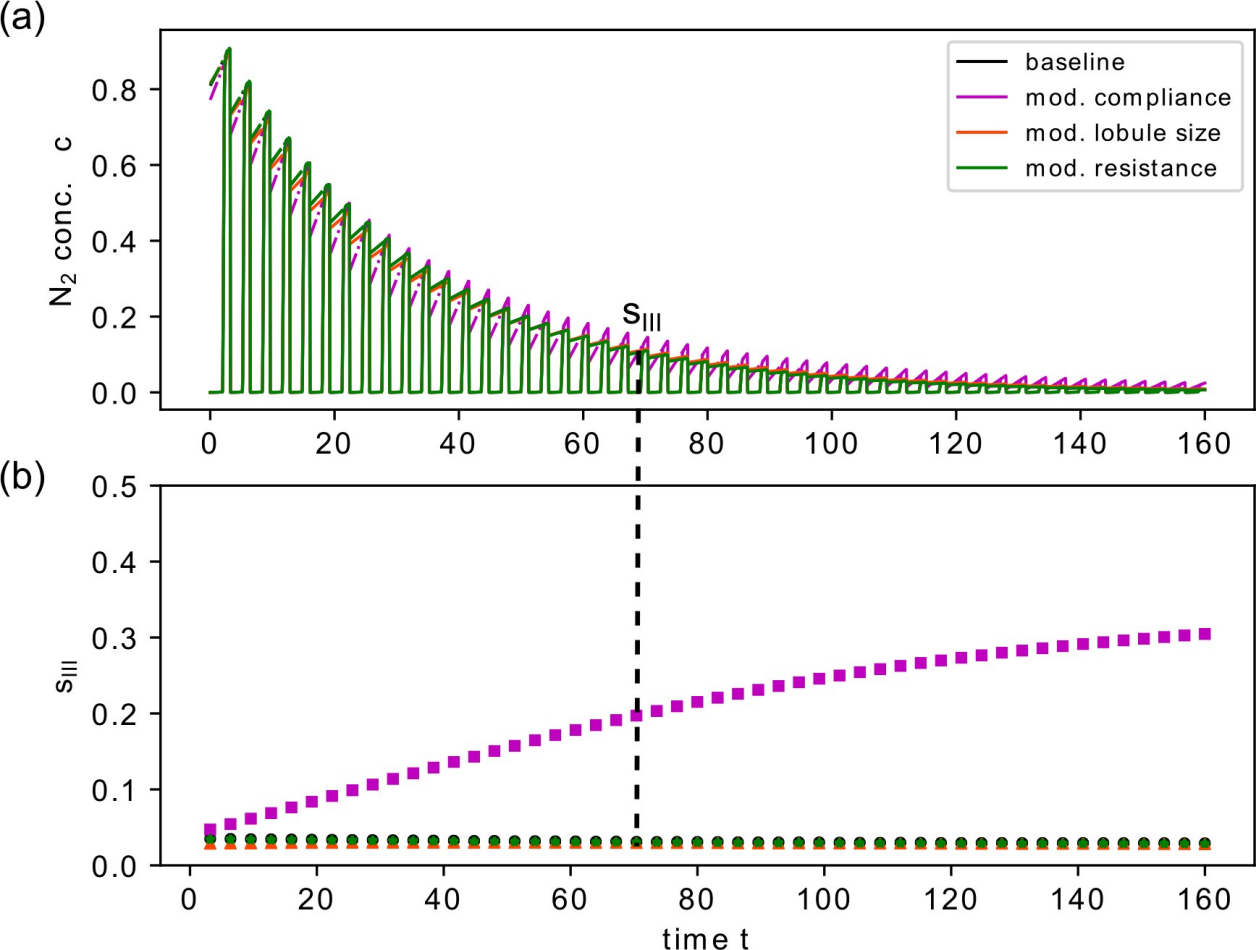
Phase III slopes s_III_ for baseline model configuration and modified trumpet lobule properties. Simulated N_2_ washout profile for baseline and different types of regional modifications with phase III slopes s_III_ indicated for each breath (a). In (b) the values for the normalized phase III slopes for each breath are shown.

The comparison of normalized phase III slopes *s*_*III*_ also clearly discriminated the compliance modifications from the other cases. In a per breath analysis, a flat plateau-like profile resulted for the baseline, as well as for the cases with altered lobule size and resistance. For regionally altered lobule compliance, however, the gas concentration increased nearly linearly in phase III. This difference is already visible from the first breath, but becomes more prominent as the washout progresses (Fig 6A and 6B). For lobular resistance and volume modifications, the phase III concentration profile remained a flat plateau during the whole washout. In the case of modified lobule compliance *s*_*III*_ increased from breath to breath. These different trends in *s*_*III*_ are illustrated in Fig 7. Increased slopes *s*_*III*_ indicate that concentration differences at airway bifurcations are increasing in phase III, such that contribution from high concentration units becomes more and more dominant. This was only the case for compliance modifications, where unequal rates of pure oxygen feed into different lobules. In case of modified volume differences, the spatial concentration distribution was not uniform, but did not change during phase III. In the next section, the relation between spatial concentration distribution in the lung and the phase III concentration profile is further discussed.

In summary, the compliance modifications had the strongest impact on gas washout in terms of non-uniform concentration decay and *s*_*III*_ values. A heterogeneity in residual lobule volume has a notable influence on the washout profile during early and later breaths, while the phase III concentration profile does not differ from the baseline. Compared to these two cases, the changes in hydrodynamic resistance have a negligible effect on gas washout profile. This is in accordance to a previous report [7].

### Regional vs. local compliance modifications

To study the capabilities of the model in reflecting the effect of different spatial distributions of structural inhomogeneities, we look again at the example of compliance modifications. Instead of applying these modifications to a subset of neighboring lobules (regional distribution), we distributed the modifications over 50% of the lobules regularly distributed in the entire lung domain (local distribution). This distribution intends to mimic lung diseases such as cystic fibrosis that do not spread in a locally organized manner [57, 58]. To this end, we performed simulations where for every other lobule the compliance was alternately reduced (*ϕ* = 0.5) or increased (*ϕ* = 1.5).

**Fig 8:**
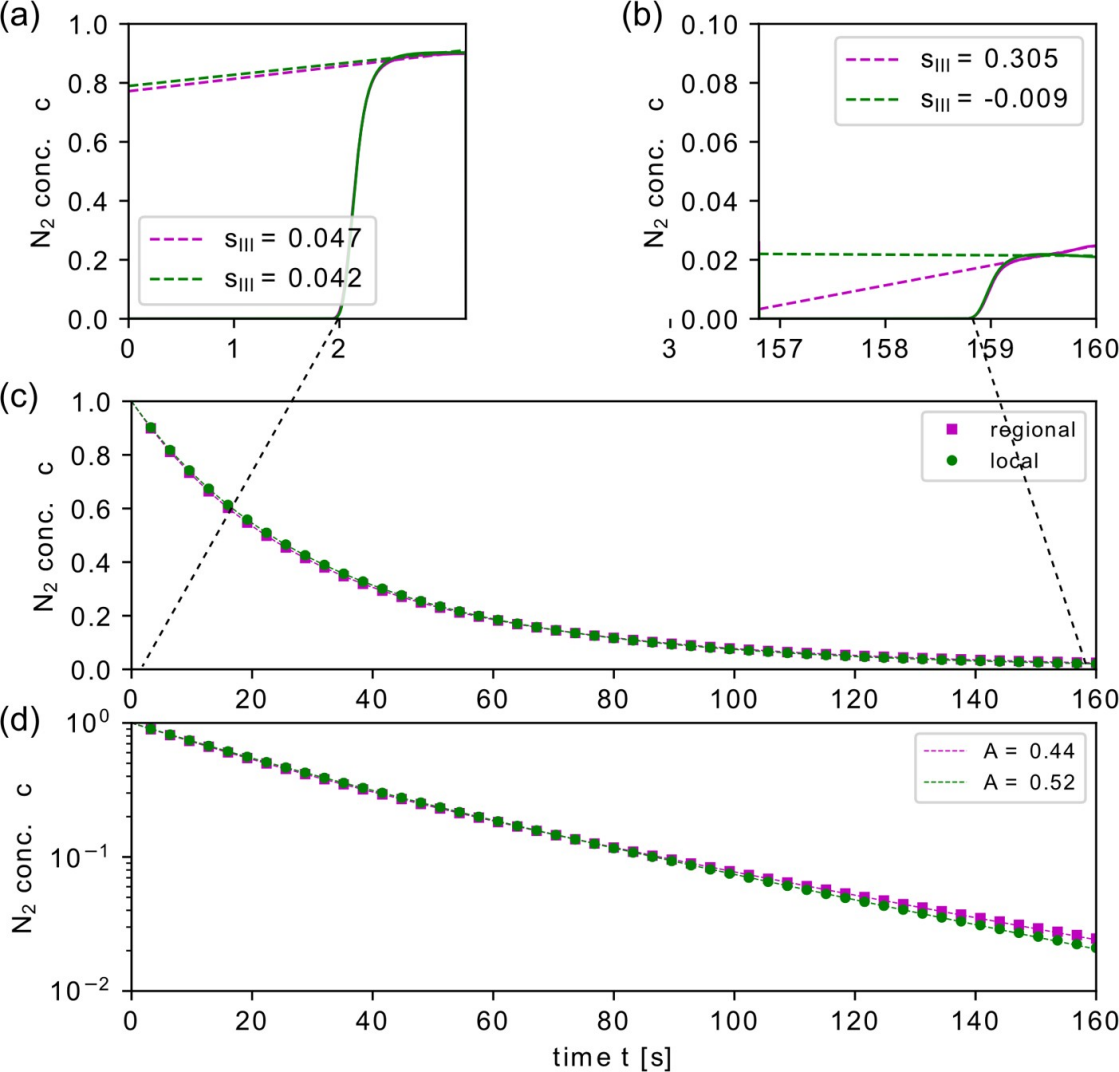
N_2_ washout for regional and local distribution of compliance modifications. Results of the simulated N_2_ gas washout for regional and local trumpet lobule compliance modifications. In both cases, the lobular compliance was altered using ϕ = 0.5, and ϕ = 1.5 each for 25% of all trumpet lobules. Normalized phase III slope s_III_ are shown for the first (a) and last (b) breath. The washout (c, d) for 50 simulated breaths, where only N_2_ end-expiratory concentrations (symbols) and the corresponding fitting function (dashed line, Eq 13) are shown for better visibility.

**Fig 9:**
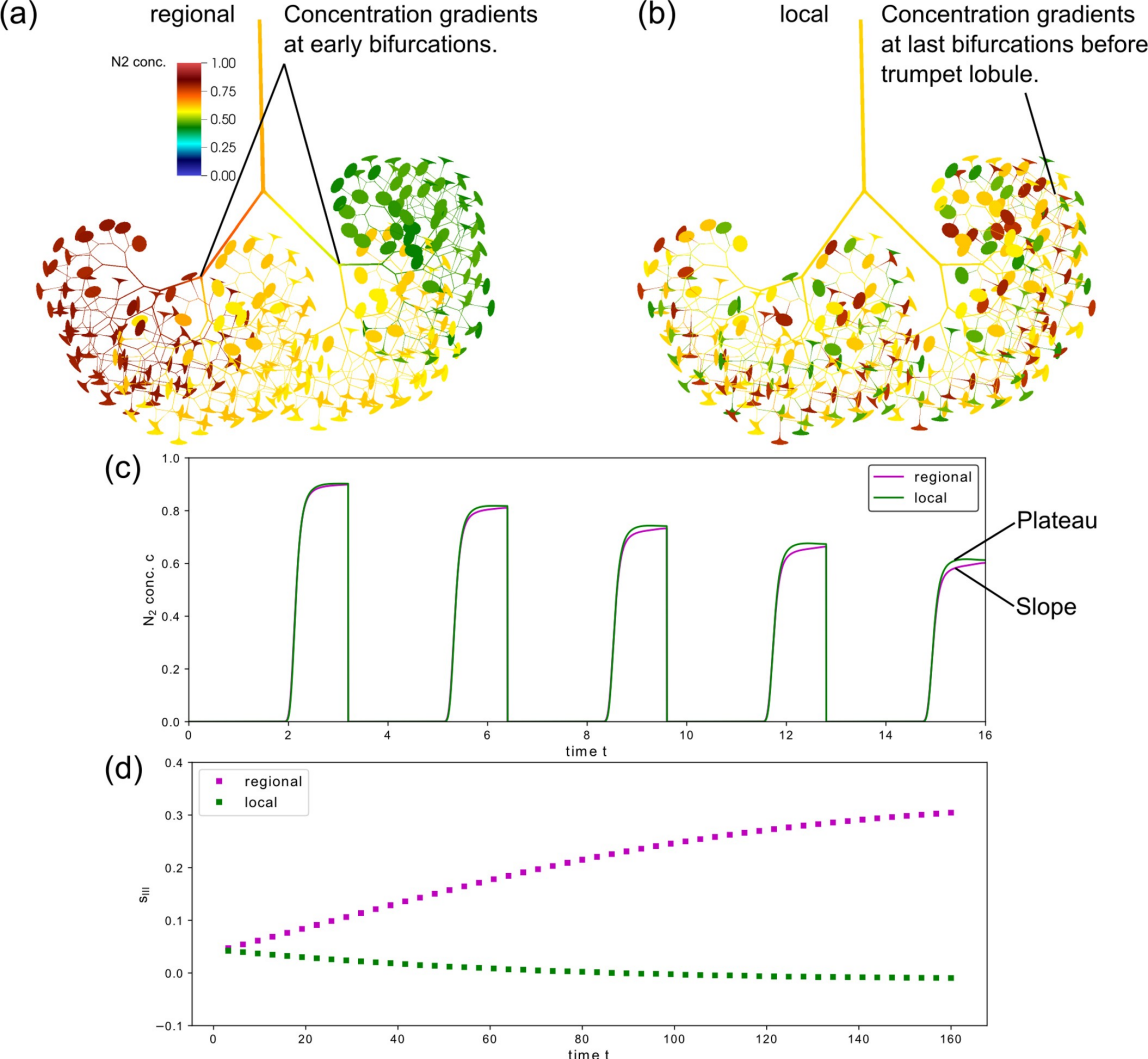
Spatial N_2_ concentration distribution after the fifth breath for regional and local type compliance modifications. Spatial concentration distributions for regional (a) and local (b) type modifications at the end of the fifth breath. In trumpet lobules with lowered compliance (ϕ = 0.5) the concentration remains high, whereas the lobules with increased compliance (ϕ = 1.5) are washed out more efficiently. In panel (c), the corresponding washout profile is shown. The different profiles in the phase III (slope vs. plateau) for regional and local type modifications correlate with the pattern of the concentrations gradients at airway bifurcations. Panel (d) shows the temporal evolution (trend) of the normalized phase III slopes s_III_ for regional and local type modifications of the lobular compliance.

Local and regional compliance modifications yielded similar results for the whole washout (Fig 8). Both caused a non-uniform decay with *A* = 0.52 (local) and *A* = 0.44 (regional). Minor differences were found in the slow and fast decay rates (*α*_1_ = 0.020 vs. 0.018 and *α*_2_ = 0.044 vs. 0.045 (local vs. regional)). However, the washout curve differed considerably with respect to phase III slope *s*_*III*_ (Fig 8A and 8B). Starting with only small differences in the first breath, *s*_*III*_ increased with every breath for regional modifications, while it decreased for local compliance modifications, reaching slightly negative values after approximately 35 simulated breaths (Fig 9D).

The spatial concentration distribution at the end of the fifth breath is depicted for both local and regional compliance modifications in Fig 9A and 9B. The lobular concentrations were clearly different for lobules with altered compliance properties. Trumpet lobules with increased compliance properties showed higher (normalized) N_2_ concentration and vice versa for reduced compliance. Furthermore, the comparison of the concentration distributions for regional and local compliance heterogeneity suggest the following mechanisms governing the phase III slopes: 1) The mixing of gas due to concentration gradients at airway bifurcations occurred at different levels: For regional modifications, the mixing took place at several generations of large airways, starting from the main bronchi to smaller conducting airways, where gas from different regions comes together, causing an increasingly non-uniform concentration distribution within the network. For local modifications, the mixing took place at already small airways and only over a few generations immediately above the lobules, which leads to a more uniform concentration distribution in the large airways. 2) Spatial concentration differences throughout the airway tree increased over time for regional modifications, while they remained approximately constant for local modifications (see S1 Video and S2 Video). This may be an explanation for the increasingly different phase III slopes (Fig 9C and 9D).

### Model for the healthy lung

In order to mimic the small degree of inhomogeneous ventilation that exists physiologically in the healthy lungs [6], we used a combination of regional modifications of lobular size (*θ* = 0.5 in two regions each accounting for 12.5% of all trumpet lobules) and lobular compliance (*ϕ* = 0.5,*ϕ* = 1.5 in two regions respectively, each accounting for 12.5% of all trumpet lobules) to approximate normal structural and mechanical inhomogeneities of the lung. The modifications parameters were determined through an iterative process: First, the total set of lobules was split in sub-regions (each 12.5%) accounting for modified lobular compliance and size. From the previously conducted modification parameter study it was known that a region with smaller lobule volume (*θ*<1) would account for faster washout during early breaths, while a region of less compliant lobule (*ϕ*<1) would cause the washout to be delayed (less efficient) towards later breaths. In a first step, the modification parameter *θ* was altered until the washout envelope of the measured and the simulated N_2_MBW curve were in agreement for the first 5–10 breaths (qualitatively, by visual inspection). Subsequently, the modification parameter *ϕ* was altered, which did not change the results from the first fitting step, until the washout envelopes also approximately matched for the last 30–50 breaths. In a MBW test simulation using this model, the lung clearance index (LCI) [1] was with 6.1 in the normal range reported in the literature [56]. S3 Video (supporting material) visualizes the evolution of the spatial concentration distribution over 50 breaths in such a model for a sinusoidal inlet flow profile. It shows the asymmetric concentration distribution due to regional differences in lobule size and lobule compliance, and how these differences evolve over time.

### Comparison between simulations and real MBW measurements

**Fig 10:**
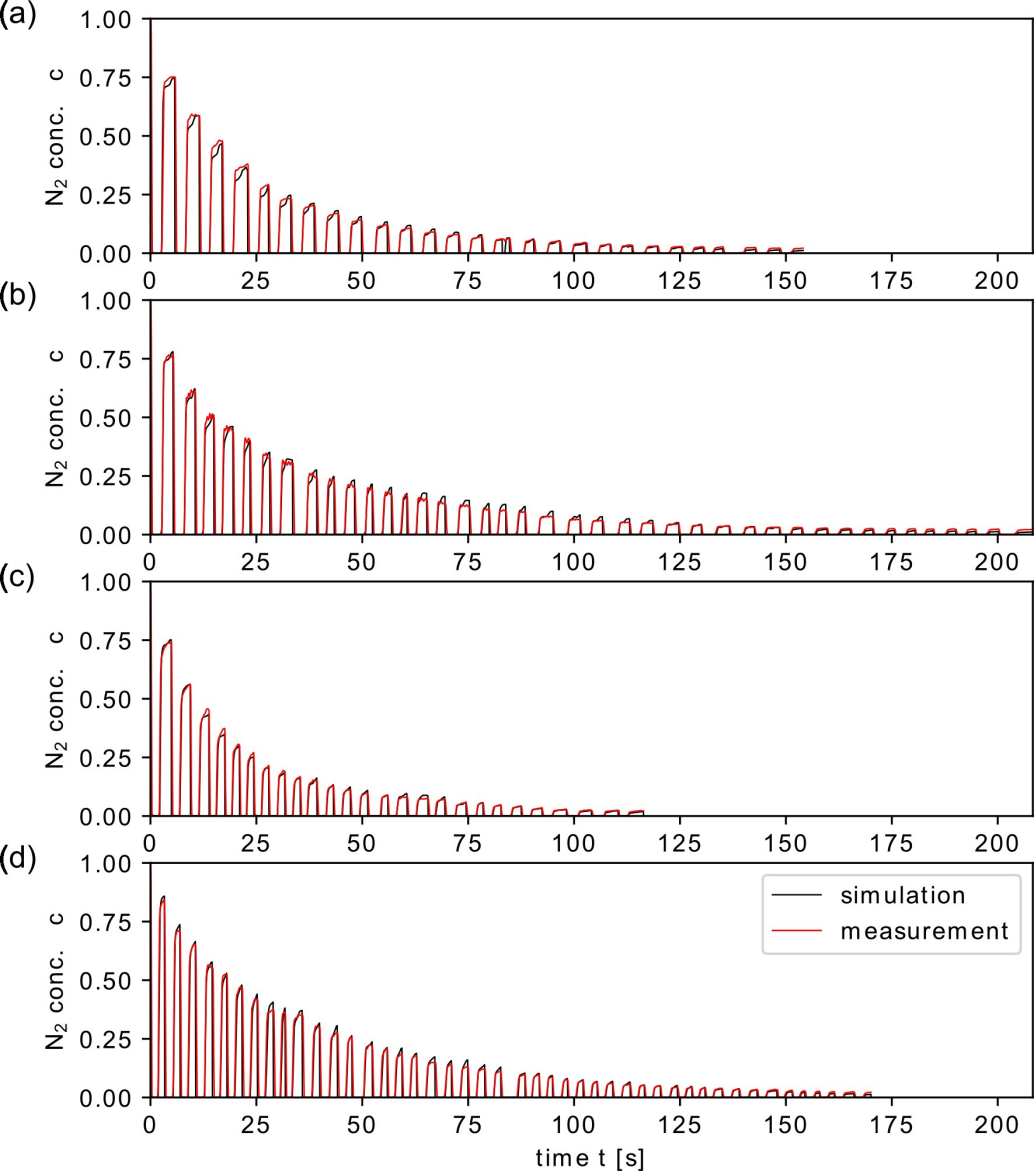
Measurements and simulation of N_2_MBW in healthy controls. Simulated N_2_ washout compared to data from N_2_MBW tests from four healthy subjects (measurements (a)—(d)). For the simulations, the inlet flow profile and the FRC as measured during the N_2_ washout test were used. To match the washout envelope in the measured data, both lobular residual volume and lobular compliance were partially modified in the lung model to mimic normal lung heterogeneity.

Demographics about the healthy subjects and lung volumes for each test simulation are provided in Table 2. For each simulation, we used the model for the healthy lung and applied the flow rate profile and the FRC as measured during the MBW test. The FRC was used to scale each lung model, using Eq 1 with the reference value FRC_W_ = 3.5 l [26]. Fig 10 and Table 3 show measured concentration curves and MBW outcomes, respectively, for N_2_MBW measured in healthy subjects together with corresponding simulations.

**Table 2:** Demographic characteristics and lung volumes for four MBW tests from healthy control adolescents.

MBW test	Subject	Age (years)	Weight (kg)	Height (cm)	sex	FRC (l)	VT (l)
#1	A	15.9	70.6	172.7	F	3.00	0.88
#2	B	17.9	50.0	169.5	M	2.85	0.67
#3	C	15.7	67.5	187.5	M	3.75	1.10
#4	C	15.7	67.5	187.5	M	3.52	0.63

M: male, F: female, FRC: functional residual capacity, VT: tidal volume. Of note, tests #3 and #4 were performed in sequence by the same subject (C).

**Table 3:** Multiple-breath washout (MBW) outcomes measured in reality and simulated in the lung model for four MBW tests from healthy control adolescents.

MBW test	LCI (TO) measured	LCI (TO) simulated	LCI washout breaths measured	LCI washout breaths simulated
#1	6.78	6.34	27	24
#2	6.37	6.22	31	30
#3	6.98	6.68	25	23
#4	7.12	6.75	43	40

LCI: lung clearance index, TO: turnovers. LCI washout breaths: number of washout breaths needed to reach the LCI

Overall, the simulated washout envelopes for the four cases were in good agreement with the experimental data. On a breath-per-breath basis, differences were more prominent. For example, end-expiratory concentration values were moderately different for several breaths. A higher variability was found during phase III. Possible reasons for this could be: the complex structural and mechanical asymmetries in the healthy lung, which are not sufficiently modelled with the types of inhomogeneities used in this study (lobular compliance, residual size, and resistance); the non-uniform breathing pattern of the healthy subjects; and measurement errors. A detailed explanation or justification of these discrepancies was beyond the scope of this study.

### Limitations of the model

The presented model has several limitations. For the sake of simplicity, several physiological and anatomical features were either idealized or neglected. The trumpet lobules include the last generations of the conducting airways, in order to keep the computational costs reasonable. We acknowledge that the acinar geometric properties are not entirely simulated in the lobule and that parameters like the homothety ratio are, in reality, not uniform throughout the airways contained in the lobule. It was beyond the scope of this study to investigate the effects of different cross-sectional development in the acinar region on the MBW washout curve. Although the dimensions of the model (airway diameter and length per generation, FRC, lobular size distribution) were derived from anatomical data, the overall geometry of the model is rather generic and not anatomically based. Spatial asymmetries between the right and the left lung were not modelled. The FRC-dependent scaling of the modeled airway tree allows using it for MBW simulations in a large FRC range, however anatomical differences between children and adults were not introduced in the model.

Pressure losses due to turbulent flow in the big airways and flow changes at the bifurcations and/or the heavily curved airways are not included in the model, as these phenomena require 3D anatomical data. However, the results in this and in previous studies [7] demonstrate that flow resistance plays a smaller role in ventilation inhomogeneity compared to differences related to the loading and constitution of respiratory units. Although there is a spatial pleural pressure gradient in real lungs, the pleural pressure was assumed to be spatially uniform, because, to our best knowledge, there is no realistic estimate for this gradient. We further assumed that the initial mechanical stress distribution is uniform between the lobules although variability should be expected in real lungs. It will be interesting to investigate the effect of this variability and effects due to gravity on future studies.

Next, regarding the model for the healthy lungs, we acknowledge that various other combinations of *θ* and *ϕ*, as well as introduction of other modifying parameters may lead to a similar MBW profile and outcomes. However, our aim was neither to mimic breathing characteristics on the individual level [59], nor to relate MBW outcomes to particular local anatomical characteristics, but to provide an example that resembles healthy lungs, based on principles of lung physiology. Finally, no specific model for the upper airways, the mouth, and the interface equipment has been used in the simulations. The additional pathway for gas transport introduced by these parts was represented by an elongation of the straight pipe representing the trachea.

### Conclusion

We presented a multi-scale model of the whole lung that simulates the gas transport and washout in conducting and acinar airways, including non-linear tissue mechanics. In order to mimic a physiological degree of ventilation inhomogeneity as described in healthy lungs, we introduced modifications in mechanical and geometrical properties on a lobular level. This study demonstrates that regional and local alterations of airway properties have different effects on the expiratory phase III in the MBW. Phase III slope profiles were notably more pronounced and sensitive to the degree of modifications for regional type modifications compared with local type modifications. Furthermore, the study revealed the different functional relations between the MBW concentration curves and airway compliance, volume and flow resistance. Increased heterogeneity of lobular compliance and residual volume correlated with a delayed washout, while heterogeneous flow resistance had a negligible impact. Finally, the simulation results are in accordance with real MBW data obtained from healthy subjects, on a qualitative level.

The model can be used to study MBW characteristics in health and disease. It offers the opportunity to understand the ventilation distribution in the healthy lung, and to investigate more profoundly MBW features that extract localized information, like the slope III analysis. By applying modifications in mechanical properties that exceed the physiological limits, the model can also mimic certain patterns of lung disease. Thus, it can be used to study the effect of such diseases on MBW concentration curves. In addition, the model may also serve as a tool to visualize gas transport in the lung during a MBW test, which could support patient education.

## Supporting information

S1 Appendix **1.The trumpet lobule model.** In this section, a geometry for the trumpet lobule cross-section is derived, which accounts for the cross-sectional growth due to exponentially increasing number of ever shorter airways, lumped into one unit (trumpet lobule). The resulting volume corresponds with the residual volume and the volume changes of a single lobule, which is prescribed by the ventilation LPM. Furthermore, a modified advection-diffusion equation is presented, which accounts for inert gas transport in the trumpet lobules with growing, time-varying cross-section. **2. Ventilation lumped parameter model.** In this section, the ventilation LPM is derived, which constitutes separate models for the non-compliant, pipe-like airways, and for the compliant (elastic) trumpet lobules. For both types of airway (unit) models the pressure-flow relation, respectively the pressure-volume relation are stated. The chosen methods for the numerical treatment of the LPM are also given in terms of an example based on a reduced size model. **3. Discretization of the transport equation.** Detailed information about the numerical treatment of the inert gas transport equation is provided. Spatial derivatives are approximated with second-order finite differences and for the temporal integration a Crank-Nicolson scheme is applied. A special focus is laid on the formulation used for an adequate treatment of the inert gas transport across airway bifurcations. Furthermore, the presented numerical scheme accounts for varying flow directions.
(PDF)

S1 Video Evolution of spatial N_2_-concentration distribution during first five breaths for regional compliance modifications. Same model modifications as in Fig 8.
(ZIP)

S2 Video Evolution of spatial N_2_-concentration distribution during first five breaths for local compliance modifications. Same model modifications as in Fig 8.
(ZIP)

S3 Video Evolution of spatial N_2_-concentration distribution during washout with 50 breaths for combined regional compliance modifications and regional size modifications. Same lung model modifications as for the model for the healthy lungs and the comparison with experimental data from healthy controls.
(ZIP)
