# Supplementary material for: A multi-scale model of gas transport in the lung to study heterogeneous lung ventilation during the multiple-breath washout test
Source: PLoS Comput Biol. 2019 Jun 17;15(6):e1007079. doi: 10.1371/journal.pcbi.1007079 (PMC6597127; doi:10.1371/journal.pcbi.1007079)
Supplement: S1 Appendix — 1.The trumpet lobule model. In this section, a geometry for the trumpet lobule cross-section is derived, which accounts for the cross-sectional growth due to exponentially increasing number of ever shorter airways, lumped into one unit (trumpet lobule). The resulting volume corresponds with the residual volume and the volume changes of a single lobule, which is prescribed by the ventilation LPM. Furthermore, a modified advection-diffusion equation is presented, which accounts for inert gas transport in the trumpet lobules with growing, time-varying cross-section. 2. Ventilation lumped parameter model. In this section, the ventilation LPM is derived, which constitutes separate models for the non-compliant, pipe-like airways, and for the compliant (elastic) trumpet lobules. For both types of airway (unit) models the pressure-flow relation, respectively the pressure-volume relation are stated. The chosen methods for the numerical treatment of the LPM are also given in terms of an example based on a reduced size model. 3. Discretization of the transport equation. Detailed information about the numerical treatment of the inert gas transport equation is provided. Spatial derivatives are approximated with second-order finite differences and for the temporal integration a Crank-Nicolson scheme is applied. A special focus is laid on the formulation used for an adequate treatment of the inert gas transport across airway bifurcations. Furthermore, the presented numerical scheme accounts for varying flow directions. (PDF) [file pcbi.1007079.s001.pdf]

1 The trumpet lobule model

The specific formulation of the transport equation (Eq. 6 and 7 in the main article) is different for pipe-like and trumpet-like airways in the lung model. In the first case, the advection velocity directly results from the solution of the lumped parameter model (LPM), solving for node pressures p_i and p_j, and the flow rate Q_{ij} between the nodes i and j, and is constant within a pipe, $u_{ij} = Q_{ij}/S_{\mathrm{ad}ij}$. Furthermore, pipes are stiff and hence $S = S_{\mathrm{ad}} = \mathrm{const.}$ Omitting the subscripts referring to nodes for briefity, the transport equation for a species concentration $c = c(x,t)$ in a pipe-like airway can be written as an advection-diffusion-equation

$$\frac{\partial c}{\partial t} + u\frac{\partial c}{\partial x} - \hat{D}\frac{\partial^2 c}{\partial x^2} = 0. \tag{1}$$

Trumpet lobule geometry

For the trumpet model representing the lobules and their peripheral airways, a model for the total cross-section of the trumpet lobule $S_{\mathrm{lb}}(x,t)$, as well as for the mean advection velocity $u_{\mathrm{lb}}(x,t)$ had to be derived. From the ventilation LPM the flow rate at the inlet of each trumpet $Q_{\mathrm{t}}(t)$ and the total volume of the trumpet $V_{\mathrm{lb}}(t) = \int_0^t Q_{\mathrm{t}}\mathrm{d}t + {V_{\mathrm{lb}}}^0$ was known. The initial volume of the trumpet lobule, $V_{\mathrm{lb}}(t=0) = {V_{\mathrm{lb}}}^0$, followed from FRC-based saling of the lung model. Assuming a uniform homothety ratio of $\kappa = 0.85$ [26] for airways lumped in a trumpet lobule, the total change of cross-section along the streamwise coordinate x can be described as

$$S(x,0) = S_{\mathrm{t}}\hat{\kappa}^{z(x)}, \quad \text{with} \quad \hat{\kappa} = 2\kappa^2 \tag{2}$$

where S_{t} is the cross-section of the terminal pipe and $z(x)$ is the generation at position x with respect to the inlet of the trumpet lobule where $z = x = 0$. Considering l_{t} to be the length of the terminal pipe, the cumulative length at generation z (with respect to the inlet of the lobule) would be $\sum_{k=1}^{z} l_{\mathrm{t}}\kappa^k$. The limes of this sum for $z \to \infty$ is

$$\lim_{z\to\infty}\sum_{k=1}^{z} l_{\mathrm{t}}\kappa^k = l_{\mathrm{t}}\kappa/(\kappa-1)) =: L. \tag{3}$$

From these relations, an expression for the generation in function of the distance to the inlet of the trumpet can be computed,

$$z(x) = \frac{\log\left[x\,\frac{\kappa-1}{\kappa\, l_{\mathrm{t}}} + 1\right]}{\log(\kappa)}. \tag{4}$$

Using equation (2) together with (4) for further treatment of the lobule model becomes a rather cumbersome task. We therefore sought a model

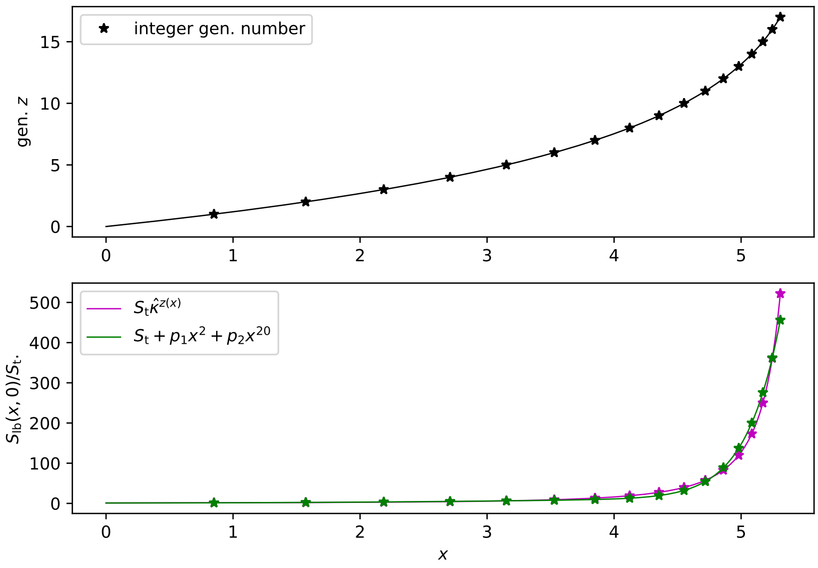

Figure 1: Above: the generation z plotted as function of the cumulative length or distance x with $l_t = d_t = unity$. The solid line indicates the function described by equation (4). Below: The cross-section as it follows from equation (2) (magenta) and the model (5) (green) where p_1 and p_2 were defined to yield the same integral as the exponential law and intersects at generation $z^\star = 5$ ($z = 0$ at trumpet inlet).

$S_{\rm lb}(x,t)$, which approximates Equation (2) but allows to derive an analytical expression for $u_{\rm lb}(x,t)$. To this end, we used a power law of the form

$$S_{\rm lb}(x,t) = p_1 x^{n_1} + p_2 x^{n_2} + S_{\rm t}, \tag{5}$$

with $n_1, n_2 = 2, 20$, where the coefficients p_1 and p_2 were defined such that the lobule cross-section $S_{\rm lb}$ intersects with Equation (2) at a chosen generation $z^\star$, and the prescribed initial volume $V_{\rm lb}^0$ of the trumpet lobule is obtained for a given length $l_{\rm lb}$ of the trumpet lobule. The coefficients were thus defined by the following linear system

$$\begin{bmatrix} x^{\star n_1} & x^{\star n_2} \\ \frac{l_{\rm lb}^{n_1+1}}{n_1+1} & \frac{l_{\rm lb}^{n_1+1}}{n_1+1} \end{bmatrix} \begin{pmatrix} p_1 \\ p_2 \end{pmatrix} = \begin{pmatrix} S^\star - S_{\rm t} \\ V_{\rm lb}^0 - S_{\rm t}\, l_{\rm lb} \end{pmatrix} \tag{6}$$

with $S^\star = S_{\rm lb}(x^\star, 0)$ and $z^\star = z(x^\star)$.

In Figure 1, a comparison between equations (2) and (5) shows a good agreement. The actual length of the trumpet model was defined based on the cumlative length $\sum_{k=1}^{z} l_{\rm t}\kappa^k$, from which $l_{\rm lb} \approx 0.98L$ follows for a lobule with $16-18$ generation of airways. The system (6) determined the initial (or end-expiratory) trumpet shape $S_{\rm lb}(x,0)$. During breathing the cross-section

widens. Therefore the first coefficient of the power law was set to be a time-depending parameter $p_1 = p_1(t)$. After the initial shape of the trumpet lobule was defined (using Equations (6)), $p_1(t)$ was updated according to the lobular volume $V_{\text{lb}}(t)$

$$V_{\text{lb}}(t) = \int_0^{l_{\text{lb}}} S_{\text{lb}}(x,t)\,\mathrm{d}x \tag{7}$$

$$\Rightarrow p_1(t) = \frac{n_1+1}{l_{\text{lb}}^{n_1+1}}\left[V_{\text{lb}}(t) - \frac{l_{\text{lb}}^{n_2+1}}{n_2+1}p_2 - S_{\text{t}} l_{\text{lb}}\right] \tag{8}$$

This determined the shape of the trumpet lobule at any time. An important feature of the model (5) is the major contribution of peripheral airway (where x is close to l_{lb}) to the overall expansion of the lung.

Modified advection-diffusion equation

An expression for the advection velocity in the trumpet model can be found introducing the 1D continuity equation for a non-constant cross-section,

$$\frac{\partial S_{\text{lb}}}{\partial t} + \frac{\partial Q_{\text{lb}}}{\partial x} = 0. \tag{9}$$

Using the model $S_{\text{lb}}(x,t)$ introduced above, the first term becomes

$$\frac{\partial S_{\text{lb}}}{\partial t} = x^{n_1}\frac{\partial p_1}{\partial t} = x^{n_1}\frac{\partial p_1}{\partial V_{\text{lb}}}\frac{\partial V_{\text{lb}}}{\partial t} = x^{n_1}\frac{n_1+1}{l_{\text{lb}}^{n_1+1}}\,Q_{\text{t}} \tag{10}$$

Integrating the continuity equation then yields an equation for the flow rate

$$\begin{aligned} Q_{\text{lb}}(x,t) &= -\int \frac{\partial S_{\text{lb}}}{\partial t}\mathrm{d}x \\ &= Q_{\text{t}}(t)\left[1 - \frac{x^{n_1+1}}{l_{\text{lb}}^{n_1+1}}\right] \end{aligned} \tag{11}$$

The term in brackets is 1 for $x = 0$ and 0 for $x = l_{\text{lb}}$, which is in agreement with the condition of zero outflow at the end of the trumpet, and follows from the constraint $Q_{\text{t}} = \mathrm{d}V_{\text{lb}}/\mathrm{d}t$. The advection velocity in the trumpet is related to the flow rate, $u_{\text{lb}}(x,t) = Q_{\text{lb}}(x,t)/S_{\text{ad}}$, where for the advection cross-section the same model as for S_{lb} is used but half the initial lobular initial volume is used for the determination of the coefficients p_1 and p_2.
The transport equation (Eq. 6 and 7 in the main article) together with the continuity equation (9) can be rearranged for non constant $S_{\text{lb}}(x,t)$ and $u_{\text{lb}}(x,t)$ yielding an advection-diffusion equation with modified advection velocity

$$\frac{\partial c}{\partial t} + \underbrace{\left[\frac{S_{\mathrm{ad}}}{S_{\mathrm{lb}}} u_{\mathrm{lb}} - \frac{\hat{D}}{S_{\mathrm{lb}}} \frac{\partial S_{\mathrm{lb}}}{\partial x}\right]}_{=\hat{u}} \frac{\partial c}{\partial x} - \hat{D} \frac{\partial^2 c}{\partial x^2} = 0 \tag{12}$$

In summary, equations (1) and (12) describe the gas transport in pipe-like airways, respectively trumpet lobules, and equations (5) and (11) together with (8) describe the dynamics of the trumpet model. The system is coupled with the ventilation LPM through the average flow velocity in each pipe and into each trumpet. The numerical solution method for the LPM and for the advection-diffusion equation are presented in the Section 2 and 3, respectively.

2 Ventilation lumped parameter model

The ventilation model consisted of coupled algebraic equations (flow balance at bifurcations of pipe-like airways) and differential equations (flow balance at junctions with compliant trumpet lobules).

Pipe-like airways

To model the pressure flow relation in pipe-like airways, we applied Womersley's theory for pulsatile flow in tubes [32]. For this case, the pressure difference between two subsequent nodes with index i and j in the network reads,

$$p_i - p_j = R_{ij} Q_{ij}, \quad \text{with} \quad R_{ij} = \mathsf{Re}\left\{ \frac{\mathsf{i}\omega\rho l_{ij}}{r_{ij}^2 \pi} \left[1 - \frac{2\mathsf{J}_1(\mathsf{i}^{3/2}\alpha)}{\mathsf{i}^{3/2}\alpha \mathsf{J}_2(\mathsf{i}^{3/2}\alpha)} \right]^{-1} \right\}. \tag{13}$$

Here, R_{ij} is the hydrodynamic resistance and Q_{ij} the flow rate in the conducting airway between nodes i and j; $\alpha = r_{ij}\sqrt{\omega\rho/\mu}$ is known as the Womersley number, $\omega = 2\pi/T_{\mathrm{B}}$ is the breathing frequency related to the breath period T_{B}; r_{ij} and l_{ij} are the radius and the length, respectively, of the conducting airway; μ is the dynamic viscosity, and ρ the density of air; and $\mathsf{i} = \sqrt{-1}$.
To save computation time in the simulations, the resistance R_{ij} for pulsatile flow is interpolated from a previously computed table for different diameters and breath periods.

Compliant trumpet lobules

For the constitutive law of the trumpet lobules, we used an exponential relation between the elastic pressure $p_{\mathrm{el}}(t)$ and the (tidal) volume of the lobule $V_{\mathrm{lb}}(t)$, which can be decomposed into a static part, V_{lb}^0 determined by

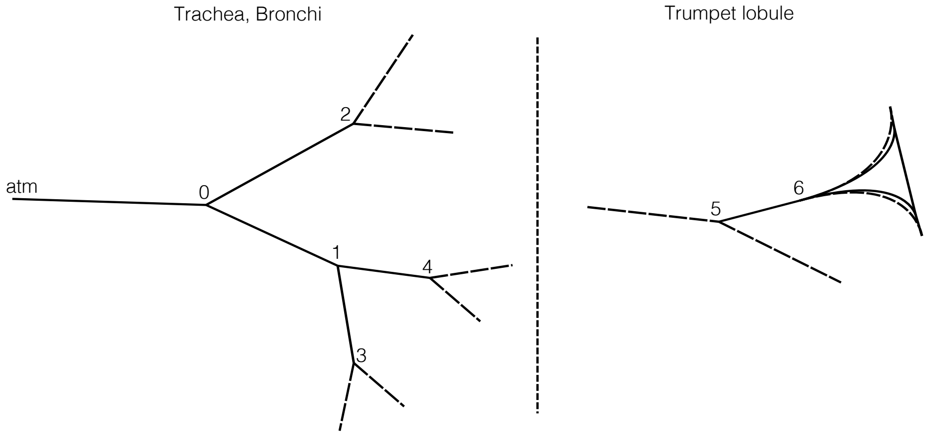

Figure 2: Reduced size network of pipe-like airways and trumpet lobule for intermediate and peripheral airways (transitional bronchioles and acini).

the number of trumpet lobules and the functional residual capacity (FRC), and a dynamic part which varies during tidal breathing $V_{\mathrm{lb}}(t) = V_{\mathrm{lb}}^0 + \tilde{V}_{\mathrm{lb}}(t)$. Additionally a pressure loss p_{diss} was introduced due to the hydrodynamic flow resistance in the lobule. Referring to Figure 2 for notations, the total pressure drop of a trumpet lobule can be stated as

$$p_6 - p_{\mathrm{pl}} = \underbrace{\beta \mathrm{e}^{\gamma V_{\mathrm{lb}}^0} \left(\mathrm{e}^{\gamma \tilde{V}_{\mathrm{lb}}} - 1\right)}_{p_{\mathrm{el}}} + \underbrace{R_{\mathrm{lb}} Q_{\mathrm{t}}}_{p_{\mathrm{diss}}}, \tag{14}$$

where p_6 is the pressure at the end of a pipe-like airway feeding into a trumpet-lobule (terminal pipe), p_{pl} is the pressure in the pleural cap, R_{lb} is the total hydrodynamic resistance within the trumpet lobule, $Q_{\mathrm{t}} = \mathrm{d}V_{\mathrm{lb}}/\mathrm{d}t$ is the flow rate at the inlet of the lobule, and V_{lb} is the lobule volume. The shape parameters β and γ were determined by the choice of a specific material law with its characteristic compliance and non-linearity (see text in the main article for more information).

Differentiation of equation (14) with respect to time and rearranging the terms yields

$$\frac{\mathrm{d}Q_{\mathrm{t}}}{\mathrm{d}t} = \frac{1}{R_{\mathrm{lb}}} \left[-\gamma \beta \mathrm{e}^{\gamma V_{\mathrm{lb}}} Q_{\mathrm{t}} + \left(\frac{\mathrm{d}p_6}{\mathrm{d}t} - \frac{\mathrm{d}p_{\mathrm{pl}}}{\mathrm{d}t} \right) \right]. \tag{15}$$

Replacing the flow rate in the terminal pipe with $Q_{\mathrm{t}} = T_{56}(p_5 - p_6)$, where T_{56} is the transmissibility (inverse of resistance) in the terminal conducting airway between nodes 5 and 6, the differential equation (15) can be written in the form

$$\frac{\mathrm{d}p_6}{\mathrm{d}t} - \frac{\mathrm{d}p_{\mathrm{pl}}}{\mathrm{d}t} - R_{\mathrm{lb}} T_{56} \left(\frac{\mathrm{d}p_5}{\mathrm{d}t} - \frac{\mathrm{d}p_6}{\mathrm{d}t} \right) = T_{56} \gamma \beta \mathrm{e}^{\gamma V_{\mathrm{lb}}} (p_5 - p_6) \tag{16}$$

Numerical solution

We applied the trapezoidal rule for the numerical integration of Equation (16).

$$
\begin{aligned}
(p_6^n - p_6^{n-1}) - (p_{\mathrm{pl}}^n - p_{\mathrm{pl}}^{n-1}) - R_{\mathrm{lb}} T_{56} \left[(p_5^n - p_5^{n-1}) - (p_6^n - p_6^{n-1}) \right] \\
= \frac{\Delta t}{2} T_{56} \gamma \beta \mathrm{e}^{\gamma V_{\mathrm{lb}}^{\star}} \left[(p_5^n + p_5^{n-1}) - (p_6^n + p_6^{n-1}) \right]
\end{aligned} \tag{17}
$$

Here, $(.)^n$ denotes variables at the new, updated, discrete time $t = n\Delta t$, with Δt the time step. The lobular volume at timestep n is estimated using an explicite Euler formulation $V_{\mathrm{lb}}^{\star} = V_{\mathrm{lb}}^{n-1} + \Delta t Q_{\mathrm{t}}^{n-1}$. For implementation purposes, Equation (17) is reformulated as

$$
-K_1 p_5^n + (1 + K_1) p_6^n - p_{\mathrm{pl}}^n = K_2 \tag{18}
$$

where the two coefficients K_1 and K_2 are computed based on available information from the former time step.

$$
K_1 = R_{\mathrm{lb}} T_{56} + \frac{\Delta t}{2} T_{56} \gamma \beta \mathrm{e}^{\gamma V_{\mathrm{lb}}^{\star}} \tag{19}
$$

$$
K_2 = -R_{\mathrm{lb}} T_{56} p_5^{n-1} + (1 + R_{\mathrm{lb}} T_{56}) p_6^{n-1} - p_{\mathrm{pl}}^{n-1} + \frac{\Delta t}{2} T_{56} \gamma \beta \mathrm{e}^{\gamma V_{\mathrm{lb}}^{\star}} \left(p_5^{n-1} - p_6^{n-1} \right) \tag{20}
$$

At bifurcations of pipe-like airways, the flow balance has to be satisfied in each time step. Referring to node 1 in Figure 2 this reads

$$
\begin{aligned}
Q_{01}^n - Q_{13}^n - Q_{14}^n = 0 \\
T_{01}(p_0^n - p_1^n) - T_{13}(p_1^n - p_3^n) - T_{14}(p_1^n - p_4^n) = 0
\end{aligned} \tag{21}
$$

subject to boundary conditions imposed at the inlet of the airway tree,

$$
T_{\mathrm{tr}}(p_{\mathrm{atm}} - p_0) = Q_{\mathrm{in}}(t) \tag{22}
$$

The flow rate profile $Q_{\mathrm{in}}(t)$ was pre-defined and constant atmospheric pressure was assumed at the inlet. T_{tr} is the transmissibility of the trachea and p_0 the pressure in the first bifurcation. Equations (18) and (21) can be stated for all bifurcation nodes and lobules. Together with the boundary conditions this yields a system of equations for the pressure values in the bifurcation nodes, p_i, and the pleural pressure, p_{pl}, which had to be solved in each time step. Thereby, for each discrete time-step, a (spatially) uniform pleural pressure was assumed. To solve the resulting system of equations, we applied an iterative method (bi-conjugate gradient stabilized solver) from the EIGEN linear algebra library (eigen.tuxfamily.org).

3 Discretization of the advection-diffusion equation

For the transport of an inert gas in the lung, two transport mechanism were considered: Advection (in this context also called convection) and molecular diffusion. The 1D-advection-diffusion equation governs the evolution of the gas concentration $c = c(x,t)$ (normalized with the nitrogen concentration in medical air, $c \in [0,1]$) in time and along the airway streamwise coordinate x:

$$\frac{\partial c}{\partial t} = -\underbrace{u\frac{\partial c}{\partial x}}_{\text{advection}} + \underbrace{D\frac{\partial^2 c}{\partial x^2}}_{\text{diffusion}} \tag{23}$$

In the lung model the velocity u was computed by means of a lumped parameter (0D) model for the ventilation of all model airway (see Section 2), and the diffusion coefficient D, given the properties of both the carrier gas and the inert tracer gas, was computed using Chapman-Enskog equation. In addition, D was modified to account for Taylor's dispersion (see [37]). For the discretization of Equation (23) the advection velocity u was assumed to be constant within a pipe-like airway. Transport phenomena related to spacial velocity gradients at the bifurcations are not considered. In trumpet lobules, the non-constant cross-section lead to a modified advection velocity $\hat{u}$ (see Section 1). Otherwise the numerical treatment was the same for pipe-like airways and trumpet lobules. A pipe or trumpet lobule was discretized into N elements and Equation (23) is evaluated on $N+1$ nodes, where N can be different for each airway. The avection-diffusion equation (23) was solved with a finite difference method applying a second order upwind scheme and a second order central scheme for the spatial discretization of the advection term and the diffusion term, respectively. For the temporal discretization a generalized Crank-Nicolsen scheme was applied. The fully discretized advection-diffusion equation reads

$$\frac{\underline{c}^{n+1} - \underline{c}^{n}}{\Delta t} = \theta(-u\underline{\underline{\mathcal{D}_1}} + D\underline{\underline{\mathcal{D}_2}})\underline{c}^{n+1} + (1-\theta)(-u\underline{\underline{\mathcal{D}_1}} + D\underline{\underline{\mathcal{D}_2}})\underline{c}^{n} + \underline{b}^{n} \tag{24}$$

Here, $\underline{c}$ is the vector containing the $N+1$ concentration values within the given airway model, $\underline{\underline{\mathcal{D}_1}}$ and $\underline{\underline{\mathcal{D}_2}}$ are finite difference operators for the first, respectively the second spatial derivatives, and $\underline{b}$ is a vector that contains boundary conditions from the neighboring pipes. The superscript n refers to the time step $t_n = n\Delta t$. Equation (24) is partly implicite due to the concentration vector considered at the new (updated) time step t_{n+1} appearing on the right-hand side. The coefficient $\theta \in [0,1]$ controls the weight of the implicite information (at t_{n+1}). For all simulations $\theta = 0.7$ was used. Re-

arranging the discretized advection-diffusion equation yields a linear system for the concentration values at the new time-step $\underline{c}^{n+1}$.

$$\underbrace{\left[1 - \Delta t\theta(-u\underline{\underline{\mathcal{D}_1}} + D\underline{\underline{\mathcal{D}_2}}))\right]}_{=:\underline{\underline{\mathcal{L}}}} \underline{c}^{n+1} = \underbrace{\left[1 + \Delta t(1-\theta)(-u\underline{\underline{\mathcal{D}_1}} + D\underline{\underline{\mathcal{D}_2}}))\right]}_{=:\underline{\underline{\mathcal{R}}}} \underline{c}^{n} + \Delta t \underline{b}^{n} \tag{25}$$

Considering the gridsize h of the 1D mesh in a pipe or trumpet-like structure, the upwind operator $\underline{\underline{\mathcal{D}_1}}$ was chosen according to the flow direction during inspiration and expiration:

$$\text{if } u \geq 0\text{:} \quad \underline{\underline{\mathcal{D}_1}} = \frac{1}{2h} \begin{bmatrix} 3 & 0 & \dots & \dots & \dots & \dots & 0 \\ -4 & 3 & 0 & \dots & \dots & \dots & \vdots \\ 1 & -4 & 3 & 0 & \dots & \dots & \vdots \\ 0 & 1 & -4 & 3 & 0 & \dots & \vdots \\ \vdots & \ddots & \ddots & \ddots & \ddots & \ddots & \vdots \\ \vdots & \dots & 0 & 1 & -4 & 3 & 0 \\ 0 & \dots & \dots & 0 & 1 & -4 & 3 \end{bmatrix} \tag{26}$$

$$\text{if } u < 0\text{:} \quad \underline{\underline{\mathcal{D}_1}} = \frac{1}{2h} \begin{bmatrix} -3 & 4 & -1 & 0 & \dots & \dots & 0 \\ 0 & -3 & 4 & -1 & 0 & \dots & \vdots \\ \vdots & \ddots & \ddots & \ddots & \ddots & \ddots & \vdots \\ \vdots & \dots & 0 & -3 & 4 & -1 & 0 \\ \vdots & \dots & \dots & 0 & -3 & 4 & -1 \\ \vdots & \dots & \dots & \dots & 0 & -3 & 4 \\ 0 & \dots & \dots & \dots & \dots & 0 & -3 \end{bmatrix}$$

The finite difference operator for the second spatial derivative is given by

$$\underline{\underline{\mathcal{D}_2}} = \frac{1}{h^2} \begin{bmatrix} -2 & 1 & 0 & \dots & \dots & \dots & 0 \\ 1 & -2 & 1 & 0 & \dots & \dots & \vdots \\ 0 & 1 & -2 & 1 & 0 & \dots & \vdots \\ \vdots & \ddots & \ddots & \ddots & \ddots & \ddots & \vdots \\ \vdots & \dots & 0 & 1 & -2 & 1 & 0 \\ \vdots & \dots & \dots & 0 & 1 & -2 & 1 \\ 0 & \dots & \dots & \dots & 0 & 1 & -2 \end{bmatrix} \tag{27}$$

Boundary and initial conditions

Bifurcation boundary conditions

Referring to the schematic in Figure 3, boundary conditions for the airway A can be stated based on the concentrations in the last nodes of airways B & C or of the first nodes in airways D & E, depending on the advection direction. The contributions of each airway B & C or D & E were weighted by the specific flow rates or cross-sections in the corresponding neighboring airways. In the following, we state the bifurcation boundary conditions for A for positive flow ($u_A > 0$), where airways B & C feed into airway A, which feeds into airways D & E, and for negative flow ($u_A < 0$), where airways D & E feed into airway A, which feeds into airways B & C. The bifurcation boundary conditions were implemented by means of ghost-nodes up-stream and down-stream of a given airway. For the considered case the ghost cell concentration values read

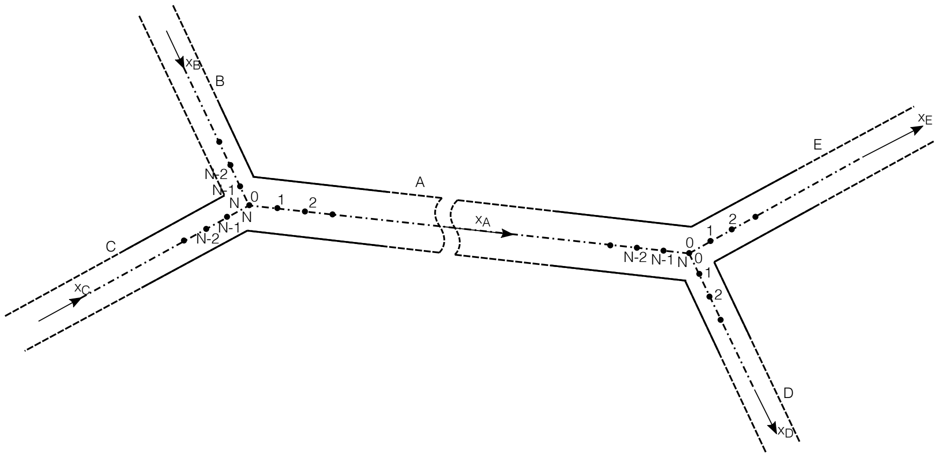

Figure 3: Bifurcation of pipe-like airways with one-dimensional grid for gas transport.

$$
\begin{aligned}
c_{A_{-1}}^{\text{adv}} &= \frac{|Q_B|}{|Q_B|+|Q_C|}c_{B_N}^{\star} + \frac{|Q_C|}{|Q_B|+|Q_C|}c_{C_N}^{\star} \\
c_{A_{-2}}^{\text{adv}} &= \frac{|Q_B|}{|Q_B|+|Q_C|}c_{B_{N-1}}^{\star} + \frac{|Q_C|}{|Q_B|+|Q_C|}c_{C_{N-1}}^{\star} \\
c_{A_{N+1}}^{\text{adv}} &= \frac{|Q_D|}{|Q_D|+|Q_E|}c_{D_1}^{\star} + \frac{|Q_E|}{|Q_D|+|Q_E|}c_{E_1}^{\star} \\
c_{A_{N+2}}^{\text{adv}} &= \frac{|Q_D|}{|Q_D|+|Q_E|}c_{D_2}^{\star} + \frac{|Q_E|}{|Q_D|+|Q_E|}c_{E_2}^{\star}
\end{aligned} \tag{28}
$$

$$
\begin{aligned}
c_{A_{-1}}^{\text{dif}} &= \frac{|S_B|}{|S_B|+|S_C|}c_{B_N}^{\star} + \frac{|S_C|}{|S_B|+|S_C|}c_{C_N}^{\star} \\
c_{A_{N+1}}^{\text{dif}} &= \frac{|S_D|}{|S_D|+|S_E|}c_{D_1}^{\star} + \frac{|S_E|}{|S_D|+|S_E|}c_{E_1}^{\star}
\end{aligned}
$$

where Q_B, Q_C, Q_D, Q_E are the flow rates in the neighboring airways of A, and $c^{\star}$ denotes a concentration value in neighboring pipe interpolated onto a grid with the same grid-size as the considered airway A. Using these values, the bifurcation boundary conditions in equation (25) were introduced with the vector $\underline{b}$ as follows

$$
\begin{aligned}
\text{if } u \geq 0: \quad \underline{b} &= \begin{pmatrix} \frac{-u_A}{2h}\left(-4c_{A_{-1}}^{\text{adv}} + c_{A_{-2}}^{\text{adv}}\right) + \frac{D}{h^2}c_{A_{-1}}^{\text{dif}} \\ \frac{-u_A}{2h}c_{A_{-2}}^{\text{adv}} \\ 0 \\ \vdots \\ +\frac{D}{h^2}c_{A_{N+1}}^{\text{dif}} \end{pmatrix} \\
\text{if } u < 0: \quad \underline{b} &= \begin{pmatrix} \frac{D}{h^2}c_{A_{-1}}^{\text{dif}} \\ \vdots \\ 0 \\ \frac{-u_A}{2h}(-c_{A_{N+1}}) \\ \frac{-u_A}{2h}\left(4c_{A_{N+1}} - c_{A_{N+2}}\right) + \frac{D}{h^2}c_{A_{N+1}} \end{pmatrix}
\end{aligned} \tag{29}
$$

Inlet and peripheral boundary conditions

At the inlet (mouth), boundary conditions of Dirichlet type with $c_0 = 0$, for inspiration, and Neumann type, $-4c_0 + 3c_1 - 1c_2 = 0$, for expiration were chosen, where c_0, c_1, and c_N refer to the normalized tracer gas concentration at the first, second and third node of the 1D grid in the trachea. At the periphery (i.e. the N-th node (last node) of the grid in the trumpet lobule),

the advection velocity is zero by definition. A zero-gradient (Neumann type) condition, $4c_N - 3c_{N-1} + 1c_{N-2} = 0$, was used to inhibit diffusive fluxes during both inspiration and expiration. The left and right-hand operator, $\mathcal{L}$ and $\mathcal{R}$ respectively, in Equation (25) were modified accordingly.

Initial condtions

In accordance with the pre-washout conditions of a typical nitrogen multiple-breath washout, i.e. uniform distribution of medical air, the concentration of the normalized tracer gas was set to $c = 1$ everywhere in the model.

Time-step and grid size

The time-step size Δt was linked to the grid-size h by means of the well known CFL condition.

$$h = \frac{u\Delta t}{CFL} \tag{30}$$

Considering a given time step Δt, the number of elements of a 1D mesh of length $l = Nh$ (in either pipe-like airways or a trumpet module) was fixed as

$$N = \texttt{floor}\left\{\frac{l\,CFL}{u\Delta t}\right\} \tag{31}$$

where $CFL = 1.0$ was used for all simulations.
If faster advection velocities (e.g. during peak inspiration or expiration) causes the number of elements to drop bellow a defined minimal number N_{min}, a time-step refinement $\Delta t/n_{\mathrm{tsr}}$ was activated, which was computed as

$$n_{\mathrm{tsr}} = \frac{u\Delta t N_{\mathrm{min}}}{l\,CFL} \tag{32}$$

The refined time-step $\Delta t/n_{\mathrm{tsr}}$ was then used for the numerical solution.
